# An Approach to Flavor Chemical Thermal Degradation Analysis

**DOI:** 10.3390/toxics12010016

**Published:** 2023-12-23

**Authors:** Michael J. Oldham, Lena Jeong, I. Gene Gillman

**Affiliations:** Juul Labs, Inc., Washington, DC 20004, USA; lena.jeong@juul.com (L.J.); gene.gillman@juul.com (I.G.G.)

**Keywords:** flavor chemical, thermal degradation, acetaldehyde, acrolein, glycidol

## Abstract

Toxicological evaluations of flavor chemicals for use in inhalation products that utilize heat for aerosol generation are complicated because of the potential effect heat may have on the flavor chemical. The objective was to develop a thermal degradation technique to screen flavor chemicals as part of a toxicological testing program for their potential use in ENDS formulations. Based upon published data for acetaldehyde, acrolein, and glycidol from ENDS products (common thermal degradants of propylene glycol and glycerin), the pyrolizer temperature was adjusted until a similar ratio of acetaldehyde, acrolein, and glycidol was obtained from a 60/40 ratio (*v*/*v*) of glycerin/propylene glycol via GC/MS analysis. For each of 90 flavor chemicals, quantitative measurements of acetaldehyde, acrolein, and glycidol, in addition to semiquantitative non-targeted analysis tentatively identifying chemicals from thermal degradation, were obtained. Twenty flavor chemicals transferred at greater than 99% intact, another 26 transferred at greater than 95% intact, and another 15 flavor chemicals transferred at greater than 90% intact. Most flavor chemicals resulted in fewer than 10–12 tentatively identified thermal degradants. The practical approach to the thermal degradation of flavor chemicals provided useful information as part of the toxicological evaluation of flavor chemicals for potential use in ENDS formulations.

## 1. Introduction

Toxicological evaluations of flavor chemicals for use as ingredients in inhalation products that utilize heat to generate an aerosol are complicated because of the potential effect heat may have on the flavor chemical. Sufficient heat may cause pyrolysis or degradation of the flavor chemical, yielding different chemicals. In cigarettes where temperatures can reach 900 °C [1], Dempsey et al. [2] have provided a historical review of these flavor chemical evaluation efforts, which have generally incorporated evaluating the pyrolysis-derived by-products [3,4]. As reported in the literature, electronic nicotine delivery systems (ENDSs) utilize substantially lower temperatures to generate an aerosol, typically under 300 °C [5,6,7,8,9,10]. However, non-temperature-regulated devices may exceed 300 °C, leading to the production of higher levels and different thermal degradation products [11]. Although the lower temperature utilized in ENDS products precludes pyrolysis, thermal degradation of flavor chemicals is still possible. Evaluation of the potential thermal degradation products from flavor chemicals used in ENDSs is consistent with a published ENDS ingredient evaluation program [5] and requirements and guidance from regulatory agencies [12,13,14].

ENDS products generally consist of a base formulation consisting of a mixture of glycerin, propylene glycol, and nicotine, although some formulations consist of only glycerin or propylene glycol and nicotine [15,16,17,18]. In addition to the base formulation, ENDS products contain various flavor chemicals. In a survey of English language websites conducted in 2014 [14], Zhu and colleagues counted 466 separate brands of e-liquid with 7764 unique e-liquid flavors. More recently, several authors have conducted studies identifying flavor chemicals in ENDS products with the majority but not all being used at low levels [19,20,21,22,23,24,25,26,27]. For example, in an examination of 20 refill fluids, the total amount of chemical flavors exceeding 0.1% occurred in only four of the refill fluids [19]. In other reports, most flavor chemicals were used at less than 1% in a brand comparison of menthol- and tobacco-flavored ENDSs [24,25].

The objective of the current work was to develop a thermal degradation technique that could be used to screen flavor chemicals as part of a toxicological testing program for their potential use in ENDS formulations.

## 2. Materials and Methods

Since the exact temperature (average or maximum) an e-liquid experiences along the coil of an ENDS product throughout a puff cannot be approximated with a single temperature [28,29] in a thermal degradation setup attached to a gas chromatograph/mass spectrometer (GC/MS), a practical approach was used. This practical approach was based upon previous work using propylene glycol showing that although different coil materials can affect the amount of carbonyl production, there appear to be similar thermal degradation processes occurring [30]. The practical approach was based upon targeted chemical analysis of several e-liquids using the Juul device [31], which is a temperature-controlled ENDS product [8,9,31]. Three chemicals (acetaldehyde, acrolein, and glycidol) were selected as indicators of the degradation of glycerin and propylene glycol to set the temperature of the thermal degradation setup attached to the GC/MS. A 60/40 percent ratio (*v*/*v*) of USP-grade glycerin and propylene glycol was used to set the analysis temperature in the thermal degradation setup. This ratio of USP-grade glycerin and propylene glycol was consistent with previous reports of commercial ENDS products [9,11,17,18,26,31]. The temperature of the GC/MS setup was tested at 275 °C and in 25 °C steps between 350 °C and 575 °C until the ratio of quantifiable acetaldehyde, acrolein, and glycidol was similar to that reported for currently marketed Juul products [31].

### 2.1. Analytical Equipment

All work was performed at a contract research organization (RTI, Laboratories, Livonia, MI, USA). This work used a GC (Agilent 6890)/MS (Agilent 5972) with a Frontier Eco-Cup LF pyrolizer (EGNPY-3030D) and a VF-wax (Agilent CP9206) 30 m × 0.25 mm column with a nominal film thickness of 1 µm and helium carrier gas (0.7 mL/min). Based upon preliminary work monitoring acetaldehyde, acrolein, and glycidol levels from 10 µL samples of USP-grade glycerin and propylene glycol in a 60/40 ratio (*v*/*v*), a temperature of 475 °C was selected to conduct the subsequent flavor chemical thermal degradation studies. The desorption time was set to 1 min. Each flavor chemical sample was placed directly in the Eco-cup to avoid volatilization that might occur in an autosampler. Quantitative analysis focused on acetaldehyde, acrolein, and glycidol using calibration curves prepared from analytical standards for each compound. The method performance was verified by acceptable recovery of 5 ng of each compound added to the USP-grade glycerin and propylene glycol mixture as samples. In addition to the quantitative determination of acetaldehyde, acrolein, and glycidol from each flavor chemical, a non-targeted semiquantitative analysis was performed using a greater than or equal to 70% match factor with the GC/MS library (Wiley 7/National Institute of Standards and Technology 05 [NIST]) to tentatively identify other potential thermal degradation products from each flavor chemical. After every 10 flavor chemical samples a quantitative standard was desorbed to demonstrate quantitative accuracy for the targeted compounds. Details of the GC separation method are in Table S1.

### 2.2. Chemical Flavors

A total of 90 flavor chemicals were evaluated using the thermal degradation setup with 5 µL for liquids or 5 µg for solids being used (Table 1). The flavor chemicals were a convenience sample and had been reported in ENDS products [19,20,21,22,23,24,25,26,27]. All flavor chemicals were food grade and compliant with Food Chemical Codex, if available, and their purity was provided by the chemical supplier.

## 3. Results

The majority of flavor chemicals tested (46) transferred intact at 95% or greater, with 20 transferring at greater than 99% intact (Table 1). Another 15 flavor chemicals tested transferred at greater than 90% intact (Table 1). Most flavor chemicals resulted in fewer than 10–13 chemicals tentatively identified due to thermal degradation, which represented less than 9% of the estimated mass (Table 1). Thermal degradation analysis of a few flavor chemicals resulted in the tentative identification of more than 30 chemicals. For the few flavor chemicals that had zero percent intact transfer, fewer than nine thermal degradation products were identified for each one (Table 1). Chromatograms for six flavor chemicals (4-vinyl guaiacol, cinnamyl isovalerate, delta-3-carene, ethyl cinnamate, methyl-2-methyl-3-furyl disulfide, and methyl benzoate) were not suitable for analysis due to late elution of the compounds as evidenced by carryover into the next injection (Table 1; Figures S1–S9).

A total of 8 of the 90 flavor chemicals produced measurable amounts of acetaldehyde, acrolein, or glycidol (Table 2). Considering that 5 µL for liquids or 5 mg for solids of each flavor chemical was analyzed, the maximum total amount of acetaldehyde, acrolein, or glycidol measured (0.33 ng/µL acetaldehyde, 1.38 ng/µL acetoin, and 1.052 for glycidol) for any flavor ingredient was a low level of thermal degradation for the targeted compounds and represented approximately 0.02–0.07% of the main intact flavor ingredient peak.

## 4. Discussion

In a few cases the percentage of intact transfer was higher than the purity of the flavor chemical (e.g., Alpha Ionone, Cital, etc.) either due to the minimum purity stated by the manufacturer and/or due to some impurities being below the limit of detection. For two flavor chemicals (2,4-Decadienal, purity 91.93%, and Melonal, purity 85.96%) the levels of the targeted chemicals and the number of tentatively identified thermal degradants may have resulted from the impurities or carrier used for these flavor chemicals, rather than from the flavor chemicals. Some of the tentatively identified thermal degradation products from the flavor chemicals (Table S2) could be of toxicological concern (e.g., pulegone identified from butyl anthranilate or ethylene oxide identified from melonal) and would guide the development of targeted chemical analysis to determine the quantitative levels of these tentatively identified thermal degradation products from flavor chemicals. Alternatively, the most prudent approach would be not to use the flavor chemical in ENDS formulation development. The toxicological evaluation of the tentatively identified chemicals or their confirmation for further toxicological evaluation of the flavor chemical is beyond the scope of this work.

One limitation in this study, as evidenced by the chromatograms for six flavor chemicals (4-vinyl guaiacol, cinnamyl isovalerate, ethyl cinnamate, delta-3-carene, methyl-2-methyl-3-furyl disulfide, and methyl benzoate) not being interpretable, is that it is likely slightly different analytical methods (e.g., different run time, columns, etc.) are required to screen a large range of flavor chemicals. A second limitation is the use of a 70% match factor to the Wiley/NIST database for chemical identification. A higher match factor (>70%) would increase confidence that the tentatively identified chemical is correct but is likely to reduce the number of tentatively identified chemicals, while using a lower match factor (<70%) would have the opposite effect, more tentatively identified chemicals with less confidence in their correct identification. Another limitation is that our use of a practical approach to set the temperature used in the GC/MS setup in this study was designed for the temperature-controlled JUUL device and would need to be adapted to other ENDS devices that have been reported to reach over 500 °C [11]. The practical approach is flexible in that the degradation products of glycerin and propylene glycol can be changed, and their ratios can be adjusted for specific ENDS devices. Finally, single-chemical thermal degradation testing cannot predict what might occur when the flavor chemical is mixed with propylene glycol, glycerin, or other flavor chemicals in an ENDS formulation [31,32], which is why this method is proposed as one screening tool for flavor chemicals, and mechanistic degradation analysis was not performed. To be clear, thermal degradation testing of flavor chemicals is not intended as a substitute for aerosol constituent measurements from the final formulation used in a final ENDS product as has been previously reported [31,33,34].

Baker and Bishop [3] evaluated the pyrolysis of 291 flavor chemicals, and Purkis et al. [4] evaluated the pyrolysis of 91 flavor chemicals, with both ramping temperatures from 300 to 900 °C in their work. The results from the current thermal degradation study were remarkably consistent with this previous pyrolysis work, especially given the dramatic differences in temperatures used between current and previous studies [3,4] (Table 3). For sixteen of the comparable flavor chemicals, the intact transfer percent was within 3% of the intact transfer percent measured in one of the previous studies; however, for the other eight flavor chemicals, differences in the intact transfer percent exceeded 5%, likely due to the temperature differences between the studies. The purity of the flavor chemicals was slightly higher in the current study compared with the purity used in one of the previous studies [3] and slightly lower compared with the other previous study [4], except for D-limonene. As anticipated due to the differences in the used temperatures, there were differences in the number of and specific degradation products identified in the current study compared with the compounds identified from the two previous pyrolysis studies (Supplemental Table S2). As noted in Baker and Bishop [3], slow or constant heat of a chemical can produce different degradation products compared with rapid heating [35].

## 5. Conclusions

In summary, a practical approach to thermal degradation testing was used as a screening tool for 90 flavor chemicals consistent with an ENDS ingredient evaluation framework [5]. Over half of the flavor chemicals transferred intact at ≥95%, with another 16% transferring intact at ≥90%. Only eight flavor chemicals produced detectable levels of acetaldehyde, acrolein, or glycidol resulting from thermal degradation. Most flavor chemicals resulted in fewer than 10–12 thermal degradation products that represented less than 9% of the mass. Overall, the practical approach to the thermal degradation of flavor chemicals provided useful information as part of the toxicological evaluation of flavor chemicals for potential use in ENDS formulations.

## Figures and Tables

**Table 1:** Chemicals evaluated via thermal degradation analysis.

Chemical Name	CAS #	FEMA #	Purity (%)	Intact Transfer %	Targeted Chemicals Identified (Yes/No)	Number of Chemicals Tentatively Identified ^1^
2,3,5,6-Tetramethyl pyrazine ^2^	1124-11-4	3237	99.90	99.45	No	7
2-Acetyl pyridine	1122-62-9	3251	99.77	98.62	No	3
2-isobutyl thiazole	18640-74-9	3134	99.54	99.34	No	9
2-isopropyl-N,2,3-trimethylbutyramide (WS-23)	51115˗67˗4	3804	99.90	83.56	No	6
2-Methyl Butyl acetate	624-41-9	3644	99.72	98.24	No	11
2-Methyl-1-Butanol	137-32-6	3998	99.94	96.34	No	9
2-Methyl-2-Pentenoic Acid	3142-72-1	3195	99.45	91.77	No	17
2,4-Decadienal, (E,E)-	25152-84-5	3135	91.93	75.78	No	25
3-Acetyl pyridine	350-03-8	3424	99.97	93.52	No	8
3-Methylcyclopentanedione (MCP)	765-70-8	2700	99.1	96.85	No	7
4-Vinyl guaiacol ^3^	7786-61-0	2675	98.06	^3^	^3^	^3^
4-(Para-Hydroxyphenol)-2-Butanone (raspberry ketone) ^2^	5471-51-2	2588	99.93	0	No	8
6-amyl-alpha pyrone	27593-23-3	3696	99.25	97.73	No	20
6-Methyl coumarin	92-48-8	2699	100	90.28	No	1
Acetoin acetate	4906-24-5	3526	99.28	99.01	Yes	7
Acetophenone ^2, 4^	98-86-2	2009	99.71	99.26	No	6
Alpha ionone ^2^	127-41-3	2594	92.66	95.47	No	18
Amyl formate	638-49-3	2068	100	78.93	No	20
Anisyl acetone	104-20-1	2672	99.78	99.08	No	7
Benzyl alcohol ^2, 4^	100-51-6	2137	99.87	100	No	0
Beta damascenone ^2, 4^	23696-85-7	3420	95.72	80.53	No	26
Beta ionone ^2, 4^	79-77-6	2595	98.9	96.96	No	21
Bis(2-methyl-3-furyl)disulfide	28588-75-2	3259	99.46	75.74	No	15
Butyl acetate ^2^	123-86-4	2174	99.78	99.53	No	5
Butyl anthranilate	7756-96-9	2181	99.71	71.45	No	44
Cinnamyl isobutyrate ^2^	103-59-3	2297	98.12	93.98	No	15
Cinnamyl isovalerate ^3^	140-27-2	2302	96.35	^3^	^3^	^3^
Cis-3-Hexenyl butyrate ((Z)-3-hexen-1-yl butyrate)	16491-64-4	3402	98.68	89.97	Yes	65
Cis-3-Hexenyl caproate	31501-11-8	3403	99.85	98.97	No	5
Cis-6-Nonenol	35854-86-5	3465	96.89	99.57	No	3
Cis-jasmone	488-10-8	3196	99.62	0	No	1
Citral ^2, 4^	5392-40-5	2303	97.62	95.52	No	26
Decanal ^2^	112-31-2	2362	98.33	91.95	Yes	26
D-limonene ^4^	5989-27-5	2633	99.45	96.7	Yes	30
Delta-3-carene ^3^	13466-78-9	3821	95.76	^3^	^3^	^3^
Diethyl malonate	105-53-3	2375	99.61	71.86	No	8
Diethyl Sebacate	110-40-7	2376	99.69	98.39	No	13
Diethyl succinate	123-25-1	2377	99.93	99.79	No	5
Dimethyl Benzyl Carbinyl Butyrate	10094-34-5	2394	99.79	79.72	No	3
Ethyl acetate ^2, 4^	141-78-6	2414	99.92	99.16	No	2
Ethyl benzoate ^2, 4^	93-89-0	2422	99.34	0	No	2
Ethyl cinnamate ^2, 3^	103-36-6	2430	99.68	^3^	^3^	^3^
Ethyl lactate ^2^	97-64-3	2440	98.88	100	No	0
Ethyl Methyl Phenyl Glycidate	77-83-8	2444	98.67	92.78	No	17
Ethyl phenyl acetate ^2^	101-97-3	2452	99.75	99.12	No	6
Ethyl-3-methylthiopropionate	13327-56-5	3343	99.95	99.86	No	3
Furfuryl thio acetate	13678-68-7	3162	99.27	94.94	Yes	15
Heptanal	111-71-7	2540	94.48	0	No	6
Hexyl alcohol ^2^	111-27-3	2567	98.55	95.99	No	14
Hexyl 2-methylbutanoate	10032-15-2	3499	99.56	90.9	No	10
Hexyl Caproate	6378-65-0	2572	99.86	98.49	No	7
Hexyl isobutyrate	2349-07-7	3172	99.64	99.51	No	7
Isoamyl phenyl acetate ^2^	102-19-2	2081	98.42	92.31	No	11
Isobutyl acetate ^2^	110-19-0	2175	99.78	88.63	No	3
L-Menthyl acetate	2623-23-6	2668	99.98	92.48	No	6
Linalool oxide ^2^	1365-19-1	3746	99.59	89.69	No	25
Melonal	106-72-9	2389	85.96	76.81	Yes	18
Methyl 3-nonenoate	13481-87-3	3710	98.08	95.37	No	21
Methyl benzoate ^2, 3, 4^	93-58-3	2683	99.62	^3^	^3^	^3^
Methyl caproate	106-70-7	2708	99.75	99.1	No	3
Methyl furfuryl disulfide	57500-00-2	3362	99.25	30.54	No	26
Methyl heptanone	110-93-0	2707	99.43	98.72	No	20
Methyl nonyl ketone	112-12-9	3093	99.82	94.42	No	5
Methylphenyl acetate ^2^	101-41-7	2733	99.99	99.61	No	2
Methyl thiobutyrate	2432-51-1	3310	99.94	99.78	No	2
Methyl-2-furoate	611-13-2	2703	99.85	99.3	No	6
Methyl-2 methyl-3-furyl disulfide ^3^	65505-17-1	3573	98.57	^3^	^3^	^3^
Milk lactone (5,6-decenoic acid)	72881-27-7	3742	87.51	91.08	No	14
N-((Ethoxycarbonyl)methyl)-p-menthane-3-carboxamide (WS-5)	68489-14-5	4309	99.64	95.05	Yes	20
Neofolione	111-79-5	2725	99.06	94.33	No	35
Neryl acetate ^2^	141-12-8	2773	98.83	91.36	No	30
N-ethyl-5-methyl-2-(1-methylethyl)cyclohexanecarboxamide (WS-3)	39711-79-0	3455	99.70	96.30	Yes	19
Nona-2-Trans, 6-Cis-Dienal	557-48-2	3377	98.94	0	No	2
Nootkatone	4674-50-4	3166	99.16	99.68	No	6
Octanone-2 (Methyl Hexyl Ketone)	111-13-7	2802	98.80	98.91	No	5
Phenylethyl phenylacetate ^2^	102-20-5	2866	99.20	96.44	No	11
Propyl acetate	109-60-4	2925	99.70	98.49	No	6
Propyl caproate	626-77-7	2949	99.91	99.88	No	4
Sulfurol (4-methyl-5-thiazole ethanol)	137-00-8	3204	99.86	43.11	No	6
Sulfuryl acetate	656-53-1	3205	99.60	0	No	9
Styrallyl Acetate (alpha-methylbenzyl acetate)	93-92-5	2684	99.75	96.85	No	24
T,T,2,4, undecadienal	30361-29-6	3422	97.75	97.32	No	12
Thiomenthone	38462-22-5	3177	97.66	87.46	No	21
Trans-2-decenal	3913-81-3	2366	97.55	98.62	No	15
Trans-2-nonenal	18829-56-6	3213	95.58	87.03	No	23
Trithioacetone	828-26-2	3475	99.55	83.57	No	20
Valencene	4630-07-3	3443	65-90	90.95	No	22
Vanillyl Ethyl Ether	13184-86-6	3815	98.32	98.95	No	8
Veratraldehyde ^2, 4^	120-14-9	3109	99.99	97.79	No	18
Whiskey lactone	39212-23-2	3803	98.62	97.64	No	6

CAS—Chemical Abstract Services; FEMA—Flavor and Extract Manufacturers Association. ^1^ Match factor greater than 70%. ^2^ Thermal degradation also evaluated by Baker and Bishop [3]. ^3^ Flavor chemicals that had unacceptable chromatography resulting in unusable data. ^4^ Thermal degradation also evaluated by Purkis et al., 2011 [4].

**Table 2:** Amount of acetaldehyde, acrolein, and glycidol resulting from thermal degradation of tested flavor chemicals.

Chemical Name	CAS #	Amount Detected (ng/µL)		
		Acetaldehyde	Acrolein	Glycidol
Acetoin Acetate	4906-24-5	<LOQ	ND	0.526
Cis-3-Hexenyl Butyrate ((Z)-3-hexen-1-yl butyrate)	16491-64-4	<LOQ	0.568	ND
D-limonene	5989-27-5	<LOQ	ND	0.158
Decanal	112-31-2	<LOQ	0.368	ND
Furfuryl thio acetate	13678-68-7	ND	ND	0.118
Melonal	106-72-9	<LOQ	0.668	ND
N-((Ethoxycarbonyl)methyl)-p-menthane-3-carboxamide (WS-5)	68489-14-5	0.166	0.688	ND
N-ethyl-5-methyl-2-(1-methylethyl)cyclohexanecarboxamide (WS-3)	39711-79-0	0.112	ND	ND

CAS—Chemical Abstract Services. LOQ—limit of quantification. ND—not detected, meaning no peaks were detected.

**Table 3:** Comparison of purity and intact transfer % of selected flavor chemicals.

Chemical Name	CAS #	Current Study		Baker and Bishop, 2004 [3]		Purkis et al. 2011 [4]	
		Purity (%)	Intact Transfer %	Purity (%)	Intact Transfer %	Purity (%)	Intact Transfer %
2,3,5,6-Tetramethyl pyrazine	1124-11-4	99.90	99.45	98	100	N/A	N/A
4-(Para-Hydroxyphenol)-2-Butanone (raspberry ketone)	5471-51-2	99.93	0	98	96.8	N/A	N/A
Acetophenone	98-86-2	99.71	99.26	98	99.8	99.9	98.7
Alpha ionone	127-41-3	92.66	95.47	90	91.8	N/A	N/A
Benzyl alcohol	100-51-6	99.87	100	99	95.2	100	94.2
Beta damascenone	23696-85-7	95.72	80.53	95	94.2	100	99.4
Beta ionone	79-77-6	98.9	96.96	97	95.4	100	100
Butyl acetate	123-86-4	99.78	99.53	99	100	N/A	N/A
Cinnamyl isobutyrate	103-59-3	98.12	93.98	97	94.0	N/A	N/A
Citral	5392-40-5	97.62	95.52	96	93.7 ^2^	100	94.4
Decanal	112-31-2	98.33	91.95	95	94.8	N/A	N/A
D-limonene	5989-27-5	99.45	96.7	N/A	N/A	98.7	100
Ethyl acetate	141-78-6	99.92	99.16	99	100	100	100
Ethyl benzoate	93-89-0	99.34	0	99	100	100	98.7
Ethyl cinnamate ^1^	103-36-6	99.68	^1^	98	98.7	N/A	N/A
Ethyl lactate	97-64-3	98.88	100	98	73.4	N/A	N/A
Ethyl phenyl acetate	101-97-3	99.75	99.12	98	98.5	N/A	N/A
Hexyl alcohol	111-27-3	98.55	95.99	98	96.3	N/A	N/A
Isoamyl phenyl acetate	102-19-2	98.42	92.31	98	94.7	N/A	N/A
Isobutyl acetate	110-19-0	99.78	88.63	98	99.8	N/A	N/A
Linalool oxide	1365-19-1	99.59	89.69	97	95.1	N/A	N/A
Methyl benzoate ^1^	93-58-3	99.62	^1^	98	99.6	100	100
Methylphenyl acetate	101-41-7	99.99	99.61	98	98.4	N/A	N/A
Neryl acetate	141-12-8	98.83	91.36	98	82.0	N/A	N/A
Phenylethyl phenylacetate	102-20-5	99.20	96.44	98	82.1	N/A	N/A
Veratraldehyde	120-14-9	99.99	97.79	98	99.6	100	100

CAS—Chemical Abstract Services; N/A = not applicable. ^1^ Flavor chemicals that had unacceptable chromatography resulting in unusable data. ^2^ Combination of E & Z-Citral.

## Data Availability

Data are contained within the article or Supplementary Material.

## Supplementary Materials

The following supporting information can be downloaded at https://www.mdpi.com/article/10.3390/toxics12010016/s1: Table S1: GC separation parameters; Table S2: Comparison of flavor ingredient degradants; Figure S1: Chromatogram (**A**) and sample mass spectra (**B**) for Cinnamyl iso-valerate (CAS# 140-27-2) demonstrating poor chromatography. One unknown peak at 23.078 min. with others eluting in subsequent runs (methyl benzoate and vinyl guaiacol injections); Figure S2: Chromatogram (**A**) and two sample mass spectra (**B**,**C**) for Methyl benzoate (CAS# 93-58-3) demonstrating poor chromatography. Injected after cinnamyl iso-valerate (CAS 140-27-2). Major peaks in this run (peaks 15 and 16 shown above) are identified as cinnamyl iso-valerate eluting late; Figure S3: Chromatogram (**A**) and mass spectra (**B**) for Vinyl guaiacol (CAS# 7786-61-0) demonstrating poor chromatography. Injected after cinnamyl iso-valerate and methyl benzoate showing major peak 2 in this run is identified as methyl benzoate eluting late compared the NIST standard mass spectra. Second set of mass spectra shows Peak 6 (RT 31.554) was identified as remaining cinnamyl iso-valerate eluting with a library match score of 95; Figure S4: Chromatogram (**A**) for delta-3-carene (CAS# 13466-78-9) demonstrating poor chromatography with no real peaks; Figure S5: Chromatogram (**A**) for ethyl cinnamate (CAS# 103-36-6) demonstrating poor chromatography with no real peaks and eluting in the next run (Methyl-2 methyl-3-furyl disulfide injection); Figure S6: Chromatogram (**A**) and mass spectra (**B**) for Methyl-2 methyl-3-furyl disulfide (CAS# 65505-17-1) demonstrating poor chromatography. Injected after ethyl cinnamate (CAS# 103-36-6). Peak 9 (RT 29.849) misidentified due to saturation. Identified as ethyl cinnamate based on comparison to reference spectrum from NIST database; Figure S7: Chromatogram (**A**) and mass spectra (**B**) for Tetramethyl pyrazine (CAS# 1124-11-4) demonstrating good chromatography. Sample mass spectra (top) compared to library match mass spectra (bottom) are below the chromatogram; Figure S8: Chromatogram (**A**) and mass spectra (**B**) for Propyl caproate (CAS# 626-77-7) demonstrating good chromatography. Sample mass spectra (top) compared to library match mass spectra (bottom) are below the chromatogram; Figure S9: Chromatogram (**A**) and mass spectra (**B**) for 2-methyl-2-pentenoic acid (CAS# 3142-72-1) demonstrating good chromatography. Sample mass spectra (top) compared to NIST match mass spectra (bottom) are below the chromatogram. Peak 14 (RT 27.105 min) misidentified due to saturation. Identified as intact 2-methyl-2-pentenoic acid based on comparison to reference spectrum from NIST database.

## References

[1] Baker R.R. (1975). Temperature variation within a cigarette combustion coal during the smoking cycle. High Temp. Sci..

[2] Dempsey R., Coggins C.R.E., Roemer E. (2011). Toxicological evaluation of cigarette ingredients. Regul. Toxicol. Pharmacol..

[3] Baker R.R., Bishop L.J. (2004). The pyrolysis of tobacco ingredients. J Anal. Appl. Pyrol..

[4] Purkis S.W., Mueller C., Intorp M. (2011). The fate of ingredients in and impact on cigarette smoke. Food Chem. Toxicol..

[5] Costigan S., Meredith C. (2015). An approach to ingredient screening and toxicological risk assessment of flavours in e-liquids. Regul. Toxicol. Pharm..

[6] Geiss O., Bianchi I., Barrero-Moreno J. (2016). Correlation of volatile carbonyl yields emitted by e-cigarettes with the temperature of the heating coil and the perceived sensorial quality of the generated vapours. Intern. J. Hyg. Environ. Health.

[7] Chen W., Wang P., Ito K., Fowles J., Shusterman D., Jaques P.A., Kumagai K. (2018). Measurement of heating coil temperature for e-cigarettes with a “top-coil” clearomizer. PLoS ONE.

[8] Black W., Alston W., Holloway G., Zhang J.T. Computational Simulation of Aerosol Generation and Temperature Regulation Performance of Nicotine Salt Pod System. Proceedings of the CORESTA Conference (Smoke—Technology Joint Study Groups).

[9] Talih S., Salman R., El-Hage R., Karam E., Karaoghlanian N., El-Hellani A., Saliba N., Shihadeh A. (2019). Characteristics and toxicant emissions of JUUL electronic cigarettes. Tob. Control.

[10] Mulder H.A., Stewart J.B., Blue I.P., Krakowiak R.I., Patterson J.L., Karin K.N., Royals J.M., DuPont A.C., Forsythe K.E., Poklis J.L. (2020). Characterization of E-cigarette coil temperature and toxic metal analysis by infrared temperature sensing and scanning electron microscopy—energy-dispersive X-ray. Inhal Toxicol..

[11] Uchiyama S., Noguchi M., Sato A., Ishitsuka M., Inaba Y., Kunugita N. (2020). Determination of thermal decomposition products generated from e-cigarettes. Chem. Res. Toxicol..

[12] European Parliament and of the Council of the European Union, Directive 2014/40/EU on the Approximation of the Laws, Regulations and Administrative Provisions of the Member States Concerning the Manufacture, Presentation and Sale of Tobacco and Related Products and Repealing Directive 2001/37/EC, Official Journal of the European Union, L127, 1–38, 3 April 2014. https://health.ec.europa.eu/publications/directive-201440eu_en.

[13] United States Food and Drug Administration, Center for Tobacco Products Guidance for Industry, Premarket Tobacco Product Applications for Electronic Nicotine Delivery Systems, March, 2023. https://www.fda.gov/media/127853/download.

[14] United States Food and Drug Administration, Center for Tobacco Products (2021). Final rule, Premarket Tobacco Product Applications and Recordkeeping Requirement. Fed. Regist..

[15] Zhu S.-H., Sun J.Y., Bonnevie E., Cummins S.E., Gamst A., Yin L., Lee M. (2014). Four hundred and sixty brands of e-cigarettes and counting: Implications for product regulation. Tob. Control.

[16] Beauval N., Antherieu S., Soyez M., Gengler N., Grova N., Howsam M., Hardy E.M., Fischer M., Appenzeller B.M.R., Goossens J.F. (2017). Chemical Evaluation of Electronic Cigarettes: Multicomponent Analysis of Liquid Refills and their Corresponding Aerosols. J. Anal. Toxicol.

[17] Oldham M.J., Zhang J., Rusyniak M.J., Kane D.B., Gardner W.P. (2018). Particle size distribution of selected electronic nicotine delivery system products. Food Chem. Toxicol..

[18] Pinto M.I., Thissen J., Hermes N., Cunningham A., Digard H., Murphy J. (2022). Chemical characterization of the vapour emitted by an e-cigarette using a ceramic wick-based technology. Sci. Rep..

[19] Hua M., Omaiye E.E., Luo E., McWhirter K.J., Pankow J.F., Talbot P. (2019). Identification of Cytotoxic Flavor Chemicals in Top-Selling Electronic Cigarette Refill Fluids. Sci. Rep..

[20] Erythropel H.C., Anastas P.T., Krishnan-Sarin S., O’Malley S.S., Jordt S.E., Zimmerman J.B. (2021). Differences in flavourant levels and synthetic coolant use between USA, EU and Canadian Juul products. Tob. Control.

[21] Jabba S.V., Erythropel H.C., Torres D.G., Delgado L.A., Woodrow J.G., Anastas P.T., Zimmerman J.B., Jordt S.-E. (2022). Synthetic cooling agents in US-marketed e-cigarette refill liquids and popular disposable e-cigarettes: Chemical analysis and risk assessment. Nicotine Tob. Res..

[22] Omaiye E.E., McWhirter K.J., Luo W., Pankow J.F., Talbot P. (2019). High-nicotine electronic cigarette products: Toxicity of JUUL fluids and aerosols correlates strongly with nicotine and some flavor chemical concentrations. Chem. Res. Toxicol..

[23] Omaiye E.E., McWhirter K.J., Luo W., Tierney P.A., Pankow J.F., Talbot P. (2019). High concentrations of flavor chemicals are present in electronic cigarette refill fluids. Sci. Rep..

[24] Omaiye E.E., Luo W., McWhirter K.J., Pankow J.F., Talbot P. (2020). Electronic cigarette refill fluids sold worldwide: Flavor chemical composition, toxicity, and hazard analysis. Chem. Res. Toxicol..

[25] Omaiye E.E., Luo W., McWhirter K.J., Pankow J.F., Talbot P. (2022). Flavour chemicals, synthetic coolants and pulegone in popular mint-flavoured and menthol-flavoured e-cigarettes. Tob. Control.

[26] Omaiye E.E., Luo W., McWhirter K.J., Pankow J.F., Talbot P. (2022). Disposable puff bar electronic cigarettes: Chemical composition and toxicity of e-liquids and a synthetic coolant. Chem. Res. Toxicol..

[27] Omaiye E.E., Luo W., McWhirter K.J., Pankow J.F., Talbot P. (2022). Ethyl maltol, vanillin, corylone and other conventional confectionery-related flavor chemicals dominate in some e-cigarette liquids labelled ‘tobacco’ flavoured. Tob. Control.

[28] Alston B. Nicotine Salt Pod System Temperature Regulation. Proceedings of the CORESTA Smoke-Techno Conference.

[29] Dibaji S.A.R., Oktem B., Williamson L., DuMond J., Cecil T., Kim J.P., Wickramasekara S., Myers M., Guha S. (2022). Characterization of aerosols generated by high-powered electronic nicotine delivery systems (ENDS): Influence of atomizer, temperature and PG:VG ratios. PLoS ONE.

[30] Saliba N.A., El Hellani A., Honein E., Salmon R., Talih S., Zeaitor J., Shihadeh A. (2018). Surface chemistry of electronic cigarette electrical heating coils: Effects of metal type on propylene glycol thermal decomposition. J. Anal. Appl. Pyrol..

[31] Chen X., Bailey P.C., Oldham M.J., Yang C., Hiraki B., Gillman I.G. (2022). Targeted characterization of the chemical composition of JUUL systems aerosol and comparison with 3R4F reference cigarettes and IQOS heat sticks. Separations.

[32] Erythropel H.C., Jabba S.V., DeWinter T.M., Mendizabal M., Anastas P.T., Jordt S.-E., Zimmerman J.B. (2019). Formation of flavorant-propylene Glycol Adducts with novel toxicological properties in chemically unstable E-cigarette liquids. Nicotine Tob. Res..

[33] Crosswhite M.R., Bailey P.C., Jeong L.N., Lioubomirov A., Yang C., Ozvald A., Jameson J.B., Gillman I.G. (2021). Non-Targeted Chemical Characterization of JUUL Virginia Tobacco Flavored Aerosols Using Liquid and Gas Chromatography. Separations.

[34] Crosswhite M.R., Jeong L.N., Bailey P.C., Jameson J.B., Lioubomirov A., Cook D., Yang C., Ozvald A., Lyndon M., Gillman I.G. (2022). Non-Targeted Chemical Characterization of JUUL-Menthol-Flavored Aerosols Using Liquid and Gas Chromatography. Separations.

[35] Baker R.R. (1979). Kinetic mechanism of the thermal decomposition of tobacco. Thermochim. Acta.

