# Supplementary material for: An Approach to Flavor Chemical Thermal Degradation Analysis
Source: Toxics. 2023 Dec 23;12(1):16. doi: 10.3390/toxics12010016 (PMC10819574; doi:10.3390/toxics12010016)
Supplement: Supplementary file 1 [file toxics-12-00016-s001.zip › toxics-2771488-supplementary.pdf]

An approach to Flavor Chemical Degradation Analysis

Supplemental Material

Michael J. Oldham[1*], Lena Jeong[1], I. Gene Gillman[1]

1. Juul Labs, Inc., Washington DC, 20004, USA

* Corresponding author: Michael J. Oldham, Michael.oldham@juul.com, Juul Labs, Inc. 1000 F St. NW, Washington, DC 20004, USA

Table S1. GC separation parameters.

**OVEN**			
Initial temp: 40 'C (On) Initial time: 2.00 min Ramps:			
#	Rate	Final temp	Final time
I	5.00	110	0.00
2	10.00	230	12.00
3	0.0	(Off)	
Post temp: 0 'C Post time: 0.00 min			
Run time: 40.00 min			
Mode: Split			
Initial temp: 230 'C (On) Pressure: 3.72 psi (On) Split ratio: 50: I			
Split flow: 34.9 mL/min Total flow: 38.4 mL/min Gas saver: Off			
Gas type: Helium			
**COLUMN 1**			
Capillary Column			
Maximum temp: 260 'C Equilibration time: 0.50 n			
Model Number: Agilent CP9206 VF-WAXms			
Max temperature: 260 'C, Nominal length: 30.0 m, Nominal diameter: 250.00 um Nominal film thickness: 1.00 um			
Mode: constant flow, Initial flow: 0.7 mL/min, Nominal init pressure: 3.72 psi, Average velocity: 30 cm/sec Inlet:			
Front Inlet, Outlet: MSD, Outlet pressure: vacuum			
**THERMAL AUX 2**			
Use: MSD Transfer Line Heater Description:			
Initial temp: 230 'C (On), Initial time: 0.00 min, # Rate Final temp Final time			
1 0.0(Off)			

Table S2. Comparison of flavor ingredient degradants.

Flavor Ingredient	CAS No.	Current Study Tentative Chemical Identification (ID match factor greater than 70) and estimated amount		Baker & Bishop, 2004		Purkis et al., 2011
		Name	Percent	Name	Percent	Number of Compounds >1% Identified
2,3,4,5-Tetramethyl pyrazine	1124-11-4	benzaldehyde, 3,4-dimethoxy	0.165	None identified		N/A
		6 unidentified compounds	0.384			
2-acetyl pyridine	1122-62-9	ethanone, 1-(2-pyridinyl)-	0.885	N/A	N/A	N/A
		b-phenylethyl formate	0.158			
		1 unidentified Compound	0.342			
2-isobutyl thiazole	18640-74-9	N,N-diethyl-1-cyclopropylethylamine	0.039	N/A	N/A	N/A
		8 unidentified compounds	0.623			
2-isopropyl-N,2,3-trimethylbutyramide (WS-23)	51115-67-4	6 unidentified compounds	16.44	N/A	N/A	N/A
2-methylbutyl acetate	624-41-9	2-butene, 2-methyl	0.12	N/A	N/A	N/A
		acetic acid, pentyl ester	0.25			
		propanoic acid, pentyl ester	0.079			
		acetic acid, pentyl ester	0.058			
		7 unidentified compounds	1.25			
2-Methyl-1-Butanol	137-32-6	beta ionone	0.618	N/A	N/A	N/A
		1-butanol	0.158			
		1-hydroxy-2-butanone	0.103			
		6 unidentified compounds	2.777			
2-methyl-2-pentenoic acid	3142-72-1	propanal	0.397	N/A	N/A	N/A
		acetic acid	0.249			
		15 unidentified compounds	7.589			
2,4-decadienal, (E,E)	25152-84-5	hexanal	1.652	N/A	N/A	N/A
		7-azanorbornene	1.162			
		hydroperoxide, pentyl	0.79			
		4,5-nonadiene	0.703			
		2,4-hexadiyne	0.443			
		benzaldehyde	0.367			
		heptanal	0.091			
		benzene, butyl-	0.081			N/A

Flavor Ingredient	CAS No.	Current Study Tentative Chemical Identification (ID match factor greater than 70) and estimated amount		Baker & Bishop, 2004		Purkis et al., 2011
		Name	Percent	Name	Percent	Number of Compounds >1% Identified
		1,1'-bicyclopropyl	0.077			N/A
		pentanal	0.055			
		15 unidentified compounds	18.801			
3-Acetyl pyridine	350-03-8	4-thiazoleethanol, 5-methyl-, acetate	5.489	N/A	N/A	N/A
		7 unidentified compounds	0.992			
3-Methylcyclopentanedione (MCP)	765-70-8	aminomethanesulfonic acid	0.851	N/A	N/A	N/A
		carbamic acid, ethyl ester	0.169			
		5 unidentified compounds	2.129			
4-vinyl guaiacol	7786-61-0	Unacceptable chromatography resulting in unusable data.		N/A	N/A	
4-(Para-Hydroxyphenol)-2-Butanone (raspberry ketone)	5471-51-2	ethanol	11.898	4-Hydroxy-4methylbenaldehyde isomer	0.8	0
		1,3-cyclopentanedione, 3-methyl	5.948	Dihydrobenzofuran	0.1	
		6 unidentified compounds	82.154	1 Unidentified compound	0.1	
6-amyl-alpha pyrone	27593-23-3	20 unidentified compounds	2.263	N/A	N/A	N/A
6-Methyl Coumarin	92-48-8	1 unidentified compound	9.725	N/A	N/A	
Acetoin acetate	4906-24-5	acetic acid	0.579	N/A	N/A	N/A
		3-hexanone	0.088			
		acetaldehyde	0.044			
		4 unidentified compounds	0.278			
Acetophenone	98-86-2	benzoic acid, 2-amino-, butyl ester	0.128	Benzoic acid	0.2	1
		azetidine, 3-methyl-3-phenyl-	0.037			
		4 unidentified compounds	0.579			
Alpha ionone	127-41-3	3-buten-2-one,4-(2,6,6-trimethyl-1-cyclohexene-1-yl)-	0.723	Trimethyldihydronaphalene +?	3.4	N/A
		1h-indene,2,3-dihydro-1,1,5,6-tetramethyl1	0.221	β-Ionone	1.3	N/A
		NApthalene, 1,2,3,4-tetrahydro-1,1,6-trimethyl	0.152	Γ-Ionone	1.2	N/A
		formic acid, ethyl ester	0.11	Trimethyltetrahydronaphalene	0.8	

Flavor Ingredient	CAS No.	Current Study Tentative Chemical Identification (ID match factor greater than 70) and estimated amount		Baker & Bishop, 2004		Purkis et al., 2011
		Name	Percent	Name	Percent	Number of Compounds >1% Identified
		methyl azide	0.068	Ethylidenemethylcylcopentene?	0.4	
		2,2-dimethylallylidenecyclopropane	0.026	6 Minor products	1.1	
		12 unidentified compounds	3.232			
Amyl formate	638-49-3	formic acid, propyl ester	8.788	N/A	N/A	N/A
		propane	0.484			
		18 unidentified compounds	11.796			
Anisyl acetone	104-20-1	cyclopropane, (1-methyl-1,2-propadienyl)-	0.074	N/A	`N/A	N/A
		6 unidentified compounds	0.846			
Benzyl alcohol	100-51-6	0 unidentified compounds	0			1
Beta damascenone	23696-85-7	butyl phthalide	6.733	(α + β + γ)-Damascone	9.2	0
		phenol, 3-methyl-	1.907	Isopropyllidenemethylcyclopentanecarboxaldehyde	0.08	
		p-xylene	0.489	Hydroxydimethylethylphenylpropane?	0.5	
		1,3-pentadiene, 2-methyl-, (E)-	0.705	5 Minor compounds	0.9	
		1-methoxy-2-methylidenecyclopropane	0.342			
		propene	0.337			
		1-butene, 2,3,3-trimethyl-	0.273			
		cyclopropane, 1,1,2,2-tetramethyl-	0.245			
		cyclopropane, 2-chloro-1,1,3-trimethyl-	0.184			
		1,3,5-cycloheptatriene	0.087			
		2-butenal	0.078			
		1,3-pentadiene, 2-methyl-	0.072			
		tocainide	0.063			
		1-hepten-4-yne, 6,6-dimethyl-	0.044			
		(E)-2-methyl-1-phenyl-1-butene	0.04			
		10 unidentified compounds	7.869			
Beta ionone	79-77-6	3-oxo-beta ionone	0.195	α-Ionone	1.1	0

Flavor Ingredient	CAS No.	Current Study Tentative Chemical Identification (ID match factor greater than 70) and estimated amount		Baker & Bishop, 2004		Purkis et al., 2011
		Name	Percent	Name	Percent	Number of Compounds >1% Identified
		6,6-dimethyl-2-vinylidenebicyclo[3.1.1]heptane	0.11	α-Ionene	0.8	
		furan, 2,3-dihydro	0.093	6 minor products, 3 unidentified	2.7	
		1-(2'-hydroxy-3',4'-dimethylphenyl)ethanone	0.052			
		3-cyclohexene-1-carboxaldehyde	0.036			
		acetic acid	0.035			
		benzaldehyde	0.023			
		14 unidentified compounds	4.498			
Bis(2-methyl-3-furyl)disulfide	28588-75-2	2-methyl-3-furanthiol	15.445	N/A	N/A	N/A
		2,4,5-tirmethyindane	0.523			
		13 unidentified compounds	8.295			
Butyl acetate	123-86-4	1-butanol	0.273	None identified		N/A
		1-butene	0.05			
		3 unidentified compounds	0.145			
butyl anthranilate	7756-96-9	cyclohexanone, 5-methyl-2-(1-methylethylidene)-	7.713	N/A	N/A	N/A
		pulegone	6.793			
		1,6-octadiene, 3,7-dimethyl-; dihydromyrcene; (+)-.beta-citronellene; citronellene; 3,7-dimethyl-1,6-octadiene;	2.509			
		pulegone; cyclohexanone, 5-methyl-2-(1-methylethylidene-, (r)-	2.170			
		cyclohexanone, 5-methyl-2-(1-methylethenyl)-	1.819			
		pulegone; cyclohexanone, 5-methyl-2-(1-methylethylidene-, (r)-	1.339			
		1,6-octadiene, 3,7-dimethyl-; dihydromyrcene; (+)-.beta-citronellene; citronellene; 3,7-dimethyl-1,6-octadiene;	1.303			

Flavor Ingredient	CAS No.	Current Study Tentative Chemical Identification (ID match factor greater than 70) and estimated amount		Baker & Bishop, 2004		Purkis et al., 2011
		Name	Percent	Name	Percent	Number of Compounds >1% Identified
		3,4-heptadiene; 1,3-diethylallene	0.389			
		bicyclo[2.2.0]hexane-1-carboxaldehyde	0.168			
		(4E)-2,3-dimethyl-2,4-nonadiene	0.131			
		syn-2-oxatricyclo[4.1.0.0(3,5)] heptane	0.115			
		33 unidentified compounds	4.101			
cinnamyl isobutyrate	103-59-3	Isothiazole, 3-methyl-	0.036	Cinnamaldehyde	2.1	N/A
		14 unidentified compounds	5.984	Cinnamylalcohol	1.0	
				Phenylpropyl acetate	0.7	
				Benzaldehyde+ propenyl benzene	0.7	
				Propenylbenzene	0.4	
				Phenyl vinyl ketone	0.3	
				1 Unidentified compound	0.8	
cinnamyl isovalerate	140-27-2	Unacceptable chromatography resulting in unusable data.		N/A	N/A	N/A
cis-3-hexenyl butyrate	16491-64-4	C2H5CH=CHCH=CH2	0.685	N/A	N/A	N/A
		(1RS, 2RS, 3RS, 4RS)-1,2:3,4-diepoxycyclopentane	0.630			
		2-butenal	0.306			
		1,3-butadiene, 2,3-dimehtyl-	0.303			
		3-butenenitrile	0.159			
		benzoic acid, 2-amino-, 2-methylpropyl ester	0.117			
		pent-4-enamide, N-(5-chloropyrid-2-yl)-	0.108			
		2,6-diamino-4-hexenoic acid	0.090			
		1,2,5,-oxadizol-3-amine, N-methyl-4-nitro-, 2-oxide	0.058			
		hydroperoxide, heptyl	0.053			
		2,4-pentadienal-	0.052			

Flavor Ingredient	CAS No.	Current Study Tentative Chemical Identification (ID match factor greater than 70) and estimated amount		Baker & Bishop, 2004		Purkis et al., 2011
		Name	Percent	Name	Percent	Number of Compounds >1% Identified
		2-propenal	0.038			
		9-oxabicyclo[6.1.0]nonan-4-one	0.032			
		52 unidentified compounds	7.396			
Cis-3-hexenyl caproate	31501-11-8	C2H5CH=CHCH=CH2	0.033	N/A	N/A	N/A
		hexanoic acid, decyl ester	0.048			
		3 unidentified compounds	0.948			
Cis-6 nonenol	35854-86-5	3 unidentified compounds	0.427	N/A	N/A	N/A
Cis-jasmone	488-10-8	(s)-2-hydroxypropanoic acid	100	N/A	N/A	N/A
Citral	5392-40-5	2-pentene, 4-bromo-	0.454	Thujone?	1.1	2
		2-propanone	0.199	Dill ether	0.8	
		furan, 2,5-dihydro-	0.187	Verbenyl ethyl ether	0.7	
		2-methyl-3-hexyne	0.172	Isoproylelidenebicyclooctane	0.7	
		1,6-octadiene, 3,5-dimethyl-, trans	0.097	Menthadienol	0.5	
		1,2-pentadiene	0.059	Methylcyclohexenone	0.1	
		2,6-octadiene, 2,6-dimethyl-	0.052	Trimethylhexadiene	0.1	
		ethanedioic acid, bis(3-methylbutyl) ester	0.049	5 Minor products	2.3	
		ethanone, 1-(2-methyl-2-cyclopenten-1-yl)-	0.045			
		furan, 2-methyl-	0.042			
		trans-2,5,5-trimethyl-1,3-hexadiene	0.031			
		cyclopropane, 1,2-dimethyl-, trans-	0.021			
		14 unidentified compounds	3.075			
Decanal	112-31-2	decanoic acid	3.944	Nonanal	2.0	N/A
		nonane	0.85	Decanol	0.6	
		1-hexene, 3,5-dimethyl-	0.34	Undecanone	0.4	
		E-11,13-tetradecadien-1-ol	0.31	Nonene	0.3	
		propanoic acid, 2-(aminooxy)-	0.212	Epoxyoctane	0.2	
		1-decene	0.099	6 Minor compounds	1.7	
		1-hexanol	0.081			
		5-decene, (E)-	0.072			

Flavor Ingredient	CAS No.	Current Study Tentative Chemical Identification (ID match factor greater than 70) and estimated amount		Baker & Bishop, 2004		Purkis et al., 2011
		Name	Percent	Name	Percent	Number of Compounds >1% Identified
		3-buten-2-one	0.07			
		methyl phenyl acetate	0.061			
		1-undecene	0.051			
		n-octan-3-ene	0.048			
		1-heptene	0.046			
		n-octenal, (E)-	0.025			
		12 unidentified compounds	1.843			
D-limonene	5989-27-5	2,5-octadiene	0.345	N/A	N/A	0
		1,2-pentadiene	0.302			
		1-butene,2-methyl-4-[(3-methyl-2-butenyl)oxy]-	0.185			
		bicyclo[2.2.0]hexane-1-carboxaldehyde	0.171			
		1-hexen-3-yne	0.138			
		1-octen-3-yne	0.057			
		4-(1,5-dimethyl-4-hexenyl)-3-cyclohexene-1-one	0.053			
		3,4-epoxy-hept-5-ene-1-yne (cis,trans 38:63)	0.05			
		butane, n-	0.028			
		2-pentyn-1-ol	0.02			
		20 unidentified compounds	1.963			
Delta-3-carene	13466-78-9	Unacceptable chromatography resulting in unusable data.		N/A	N/A	N/A
Diethyl malonate	105-53-3	formic acid, ethyl ester	3.654	N/A	N/A	N/A
		7 unidentified compounds	24.487			
Diethyl sebacate	110-40-7	3,4-epoxy-hept-5-ene-1-yne (cis,trans 38:62)	0.019	N/A	N/A	N/A
		hexanoic acid	0.013			
		11 unidentified compounds	1.584			
Diethyl succinate	123-25-1	5 unidentified compounds	0.210	N/A	N/A	N/A

Flavor Ingredient	CAS No.	Current Study Tentative Chemical Identification (ID match factor greater than 70) and estimated amount		Baker & Bishop, 2004		Purkis et al., 2011
		Name	Percent	Name	Percent	Number of Compounds >1% Identified
Dimethyl benzyl carbinyl butyrate	10094-34-5	3 unidentified compounds	20.279	N/A	N/A	N/A
Ethyl acetate	141-78-6	acetic acid	0.377	None identified		0
		1 unidentified compound	0.464			
Ethyl benzoate	93-89-0	Hexanoic acid, 4-hexen-1-yl ester, (Z)-	65.449	None identified		1
		1 unidentified compound	34.551			
Ethyl cinnamate	103-36-6	Unacceptable chromatography resulting in unusable data.		Benzaldehyde	0.7	N/A
				Styrene	0.4	
				Methyl cinnamate	0.2	
Ethyl lactate	97-64-3	0 unidentified compounds		Acetol acetate?	N/A	N/A
				Acetic acid?	N/A	
				4 Unidentified compounds	N/A	
Ethyl methyl phenyl glycidate	77-83-8	ethanone, 1-phenyl	0.136	N/A	N/A	N/A
		benzeneacetaldehyde	0.111			
		15 unidentified compounds	6.97			
Ethyl phenyl acetate	101-97-3	benzeneacetic acid	0.29	Ethylbenzoyl formate	0.8	N/A
		2-butanone,4-(4-methoxyphenyl)-	0.213	Benzaldehyde	0.7	
		benzenepropanenitrile, beta -oxo-	0.099			
		benzoic acid, ethyl ester	0.049			
		2 unidentified compounds	0.23			
ethyl-3-methylthiopropionate	13327-56-5	3 unidentified compounds	0.136	N/A	N/A	N/A
Furfuryl thio acetate	13678-68-7	cinnamyl iso valerate	2.054	N/A	N/A	N/A
		2-furanmethanethiol	1.072			
		6-(diethoxyphosphinyl)-2-methylcyclohex-2-en-1-one	0.242			
		2,6-dimethyl pyrazine	0.075			
		11 unidentified compounds	1.615			
Heptanal	111-71-7	1-pentene, 2,3-dimethyl	74.34	N/A	N/A	N/A
		2,6-nonadienal, (e,z)-	4.88			
		cis-jasmone	2.465			
		3 unidentified compounds	18.316			

Flavor Ingredient	CAS No.	Current Study Tentative Chemical Identification (ID match factor greater than 70) and estimated amount		Baker & Bishop, 2004		Purkis et al., 2011
		Name	Percent	Name	Percent	Number of Compounds >1% Identified
Hexyl alcohol	111-27-3	1-octanol	1.148	Hexanal	3.5	N/A
		1-butanol, 2-ethyl	0.596	Ethyl hexanoate	0.1	
		3-octanol	0.523	Undecane	0.1	
		1-butanol	0.127			
		n-nonane	0.124			
		1-hexanol, 2-ethyl	0.087			
		1-pentanol	0.087			
		1-butanol, 3-methyl	0.085			
		cis-jasmone	0.079			
		5 unidentified compounds	1.153			
Hexyl 2-Methylbutanoate	10032-15-2	formic acid, ethyl ester	2.540	N/A	N/A	N/A
		9 unidentified compounds	6.563			
Hexyl caproate	6378-65-0	7 unidentified compounds	1.511	N/A	N/A	N/A
Hexyl isobutyrate	2349-07-7	propanoic acid, 2-methyl-, octyl ester	0.155	N/A	N/A	N/A
		2-methoxyethoxy-2-methylethyl 2-(2'-hydroxy-1'-methyl)ethoxy]-1-methylethyl ether	0.006			
		benzoic acid	0.039			
		4 unidentified compounds	0.291			
Isoamyl phenyl acetate	102-19-2	benzeneacetic acid	1.889	Benzaldehyde	3.8	N/A
		1-butanol, 3-methyl-	1.716	Ethyl phenylacetate	0.9	
		1-propanone, 2-methyl-1-phenyl-	1.664	Methylbutanol	0.4	
		1-butene, 3-methyl-	0.241	Phenyl benzoate	0.2	
		b-phenylethyl formate	0.18			
		benzaldehyde	0.049			
		benzeneacetic acid, ethyl ester	0.046			
		4 unidentified compounds	1.912			
Isobutyl acetate	110-19-0	cis-jasmone	10.5	n-Butyl acetate	0.1	N/A
		2 unidentified compounds	0.88	1 Unidentified compound	0.1	

Flavor Ingredient	CAS No.	Current Study Tentative Chemical Identification (ID match factor greater than 70) and estimated amount		Baker & Bishop, 2004		Purkis et al., 2011
		Name	Percent	Name	Percent	Number of Compounds >1% Identified
L-menthyl acetate	2623-23-6	cyclohexene, 4-menthyl-1-(1-methylethyl)-	3.486	N/A	N/A	N/A
		cyclohexene, 4-menthyl-1-(1-methylethyl)-	2.797			
		acetic acid	1.108			
		2-butanone, 4-(4-methoxyphenyl)-	0.078			
		1-isopropenyl-1-(1-propenyl)cyclohexane	0.03			
		1 unidentified Compound	0.019			
Linalool Oxide	1365-19-1	4-ethyl-2,5,6-trimethylpyrimidine	1.295	Methylvinylbutyrolactone	2.3	N/A
		(Z)-2-fluoro-3-phenylpropenal	0.752	Methylcaprolactone	0.8	
		1,2-Dimethyl cyclopropene	0.011	Diethylmethoxycyclopropane	0.5	
		22 unidentified compounds	8.251	Methylheptenone	0.3	
				Pentylacetyl propanoate	0.1	
				3 Unidentified compounds	0.9	
Melonal	106-72-9	6-methyl-5-hepten-2-one	14.476	N/A	N/A	N/A
		1,3-butadiene, 2,3-dimethyl-	5.920			
		2h-pyran-2-one, tetrahydro-6,6-dimethyl-	0.371			
		2-butenal, 2-ethenyl-	0.268			
		cyclopropane	0.137			
		1,3-butadiene, 2-methyl-	0.116			
		cis-1-acetyl-2-methyl-3-cyclohexene	0.085			
		butane, n-	0.072			
		2-butenal	0.066			
		ethylene oxide	0.031			
		8 unidentified compounds	1.643			
Methyl-3-nonenoate	13481-87-3	d-limonene	0.206	N/A	N/A	N/A
		2,4-hexadienal	0.051			
		5-methyl furfural	0.028			
		18 unidentified compounds	4.341			

Flavor Ingredient	CAS No.	Current Study Tentative Chemical Identification (ID match factor greater than 70) and estimated amount		Baker & Bishop, 2004		Purkis et al., 2011
		Name	Percent	Name	Percent	Number of Compounds >1% Identified
Methyl benzoate	93-58-3	Unacceptable chromatography resulting in unusable data.		Ethyl benzoate	0.4	0
Methyl caproate	106-70-7	cis-jasmone	0.695	N/A	N/A	N/A
		pentanoic acid, methyl ester	0.114			
		1 unidentified Compound	0.119			
Methyl furfuryl disulfide	57500-00-2	2-cyclopenten-1-one	9.013	N/A	N/A	N/A
		disulfide, dimethyl	3.848	N/A	N/A	N/A
		1,2,4-trithiolane	2.909			
		furan, 2,2'vinylenedi-	2.887			
		2(1H)-pyridinethione, 3-ethoxy-6-methyl-	1.671			
		methanethiol	0.923			
		cyclopropanal, methylene	0.863			
		19 unidentified compounds	47.339			
Methyl heptenone	110-93-0	2-(N-methylimino)propanenitrile	0.102	N/A	N/A	N/A
		benzoic acid, ethyl ester	0.096			
		2-propanone	0.083			
		2-azido-5-methyl-1,4-benzoquinone	0.066			
		1,3-butadiene, 2,3-dimethyl-	0.061			
		2-butene-1,4-diol, 2,3-dibromo-	0.042			
		2-hexyn-l-ol	0.037			
		13 unidentified compounds	0.797			
Methyl Nonyl Ketone	112-12-9	5 unidentified compounds	5.579	N/A	N/A	N/A
Methylphenyl acetate	101-41-7	ethanone, 2-hydroxy-1-phenyl	0.056	Ethyl phenylacetate	0.9	N/A
		1 unidentified Compound	0.334	Phenethyl alcohol	0.7	
Methyl thiobutyrate	2432-51-1	2 unidentified compounds	0.217	N/A	N/A	N/A
Methyl-2-furoate	611-13-2	6 unidentified compounds	0.704	N/A	N/A	
Methyl-2-methyl-3-furyl disulfide	65505-17-1	Unacceptable chromatography resulting in unusable data.		N/A	N/A	N/A
Milk lactone (5,6-decenoic acid)	72881-27-7	2(3H)-furanone, 5-acetyldihydro-	1.816	N/A	N/A	N/A
		trans-2-ethyl-2-hexen-1-ol	0.103			

Flavor Ingredient	CAS No.	Current Study Tentative Chemical Identification (ID match factor greater than 70) and estimated amount		Baker & Bishop, 2004		Purkis et al., 2011
		Name	Percent	Name	Percent	Number of Compounds >1% Identified
		12 unidentified compounds	7.001			
N-((Ethoxycarbonyl)methyl)-p-menthane-3-carboxamide (WS-5)	68489-14-5	Cyclohexene, 4-methyl-1-(1-methylethyl)-	0.17	N/A	N/A	N/A
		1,3,5-Hexatriene, (E)-	0.06			
		1,3-pentadiene, 3-methyl, (E)-	0.02			
		Ethyl Alcohol	0.02			
		16 unidentified compounds	4.69			
Neofolione	111-79-5	cyclopropane carboxaldehyde	0.215	N/A	N/A	N/A
		2-heptanone	0.048			
		hydroperoxide, 1-methylpentyl	0.04			
		9-oxabicyclo[6.1.0]nonan-4-one	0.039			
		furan, 2,5-dihydro-3-methyl	0.034			
		2-nonenal	0.029			
		1,6-octadiene, 3,7-dimethyl	0.024			
		6-carbomethoxybicyclo[4.3.0]non-2-en-4one	0.024			
		cyclopentene, 3-ethyl	0.023			
		2-pentenoic acid, methyl ester	0.019			
		methyl azide	0.018			
		24 unidentified compounds	5.156			
Neryl acetate	141-12-8	linayly acetate	0.882	Pulegone	4.9	N/A
		nopinen	0.429	Liminene	2.8	
		acetic acid	0.173	B-Myrcene	1.9	
		linayly acetate	0.153	B-Ocimene	1.6	
		Z-beta -ocimene	0.108	B-Pinene	1.1	
		25 unidentified compounds	6.897	A-Terpinolene	0.9	
				δ-3-Carene	0.8	
				16 minor products, 2 unidentified	3.7	
N-ethyl-5-methyl-2-(1-methylethyl)cyclohexanecarboxamide (WS-3)	39711-79-0	Cyclohexene, 4-methyl-1-(1-methylethyl)-	0.11	N/A	N/A	N/A
		Cyclohexene, 6-(1-methylethyl)-	0.05			

Flavor Ingredient	CAS No.	Current Study Tentative Chemical Identification (ID match factor greater than 70) and estimated amount		Baker & Bishop, 2004		Purkis et al., 2011
		Name	Percent	Name	Percent	Number of Compounds >1% Identified
		1,3-Pentadiene, 2-methyl-	0.01			
		16 unidentified compounds	3.52			
Nona-2-Trans, 6-Cis-Ddienal	557-48-2	benzeneacetic acid, ethyl ester	70.974	N/A	N/A	N/A
		2-butanone, 4-(4-methoxyphenly)-	29.026			
Nootkatone	4674-50-4	veratraldehyde	0.076	N/A	N/A	N/A
		pyrazine, tetramethyl	0.074			
		4 unidentified compounds	0.167			
Octanone-2 (Methyl Hexyl Ketone)	111-13-7	2-octanol	0.812	N/A	N/A	N/A
		butane	0.049			
		3 unidentified compounds	0.225			
Phenylethyl phenylacetate	102-20-5	benzeneacetic acid, phenylmethyl ester	0.734	Phenethyl alcohol	2.9	N/A
		bicyclo[4.2.0]octa-1,3,5-triene	0.29	Benzaldehyde	4,8	
		benzeneacetic acid, 2-phenylethyl ester	0.188	Acetophenone?	3.5	
		benzeneethanol	0.16	Ethyl phenylacetate	2.0	
		benzaldehyde	0.068	Styrene	1.2	
		formic acid, 2-phenylethyl ester	0.027	Phenol	0.5	
		benzeneacetic acid, methyl ester	0.021	Hyacinthin	0.5	
		benzene, methyl-	0.018	Dibenzyl + Benzofuran	0.2	
		3 unidentified compounds	2.056	Toluene	0.1	
				8 Minor products	2.2	
Propyl acetate	109-60-4	1-propanol	0.55	N/A	N/A	N/A
		cis-jasmone	0.129			
		acetic acid	0.121			
		propene	0.09			
		2 unidentified compounds	0.624			
Propyl caproate	626-77-7	z-citral	0.03	N/A	N/A	N/A
		1-heptyne	0.017			
		2 unidentified compounds	0.073			

Flavor Ingredient	CAS No.	Current Study Tentative Chemical Identification (ID match factor greater than 70) and estimated amount		Baker & Bishop, 2004		Purkis et al., 2011
		Name	Percent	Name	Percent	Number of Compounds >1% Identified
Sulfurol (4-methyl-5-thiazole ethanol)	137-00-8	6 unidentified compounds	56.893	N/A	N/A	N/A
Sulfuryl acetate	656-53-1	9 unidentified compounds	100.000	N/A	N/A	N/A
Styrallyl acetate (alpha-methylbenzyl acetate)	93-92-5	cis-jasmone	0.613	N/A	N/A	N/A
		ethane, 1,2-diethoxy-	0.383			
		(E)-dec-2-en-4-yNAl	0.326			
		7-methylocta-1,3(Z),5(E)-triene	0.219			
		cis-jasmone	0.202			
		1-(m-ethylphenyl)ethanol	0.152			
		4-pentenal	0.095			
		1-octen-3-yne	0.074			
		2-cyclopenten-1-one, 2-methyl-	0.056			
		cyclooctene	0.045			
		1,3-pentadiene, (E)-	0.042			
		2-butenal	0.032			
		bicyclo[4.2.0]octa-1,3,5-triene	0.026			
		propanal	0.023			
		10 unidentified compounds	0.86			
T,T,2,4, undecadienal	30361-29-6	benzoic acid, methyl ester	1.080	N/A	N/A	N/A
		11 unidentified compounds	1.601			
Thiomenthone	38462-22-5	cyclohexanone, 3-m,ethyl- (r)-	4.537	N/A	N/A	N/A
		pulegone	2.406			
		2,6-octadiene, 2,6-dimethyl	1.205			
		hydrogen sulfide	0.236			
		methozxy phenyl butanone	0.061			
		cyclohexanone, 5-methyl-2-(1-methylethyl)-,cis-	0.036			
		1,3,5-hexatriene, 3-methyl- (e)-	0.021			
		14 unidentified compounds	4.038			
Trans-2-decenal	3913-81-3	15 unidentified compounds	1.383	N/A	N/A	N/A
Trans-2 nonenal	18829-56-6	2-noneoic acid	5.321	N/A	N/A	N/A

Commented [LJ(1]: Nominal mass matches to major peak (98.987%), but no reference EI spectrum found. Could we predict fragmentation?

Flavor Ingredient	CAS No.	Current Study Tentative Chemical Identification (ID match factor greater than 70) and estimated amount		Baker & Bishop, 2004		Purkis et al., 2011
		Name	Percent	Name	Percent	Number of Compounds >1% Identified
		5h-tetrazolo[1,5-a]azepine, 6,7,8,9-tetrahydro-	0.514			
		propane	0.208			
		cyclopropane carboxaldehyde	0.067			
		19 unidentified compounds	6.855			
Trithioacetone	828-26-2	cis-jasmone	1.392	N/A	N/A	N/A
		hydrogen sulfide (h2s)	0.326			
		thiophene, 2-ethyl-	0.114			
		trans-1,4-hexadiene	0.064			
		cyclopropane	0.054			
		ditehylamine-d1	0.04			
		14 unidentified compounds	14.678			
Valencene	4630-07-3	(1S(*),5S(*),8S(*))-5-methyltricyclo[6.3.0.0(1,5)]undec2-en-one	5.897	N/A	N/A	N/A
		cyclohexanone	0.415			
		3-butenoic acid	0.053			
		bis(3-methylbutyl) fluorene-2,7-disulfonate	0.025			
		18 unidentified compounds	2.656			
Vanillyl ethyl ether	13184-86-6	tropone	0.207	N/A	N/A	N/A
		7 unidentified compounds	0.840			
Veratraldehyde	120-14-9	benzene, 1,2-dimethoxy	0.278	Veratrole	0.2	0
		benzaldehyde, 4-methyl	0.031	Methyl veratrate	0.1	
		16 unidentified compounds	1.905	Dimethyl oxalate	0.1	
Whiskey lactone	39212-23-2	2-hexyl hydroperoxide	0.062	N/A	N/A	N/A
		5 unidentified compounds	2.301			

CAS – Chemical Abstract Services; N/A = not applicable.

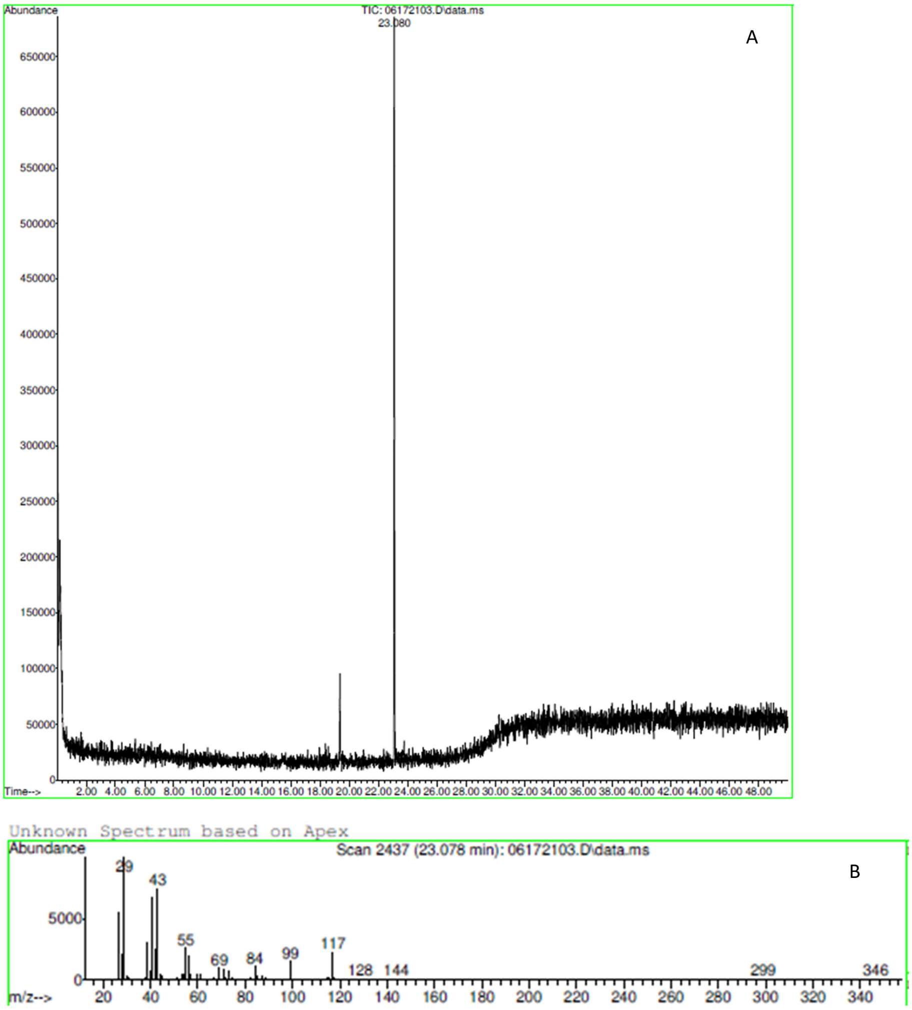

Figure S1. Chromatogram (A) and sample mass spectra (B) for Cinnamyl iso-valerate (CAS# 140-27-2) demonstrating poor chromatography. One unknown peak at 23.078 min. with others eluting in subsequent runs (methyl benzoate and vinyl guaiacol injections).

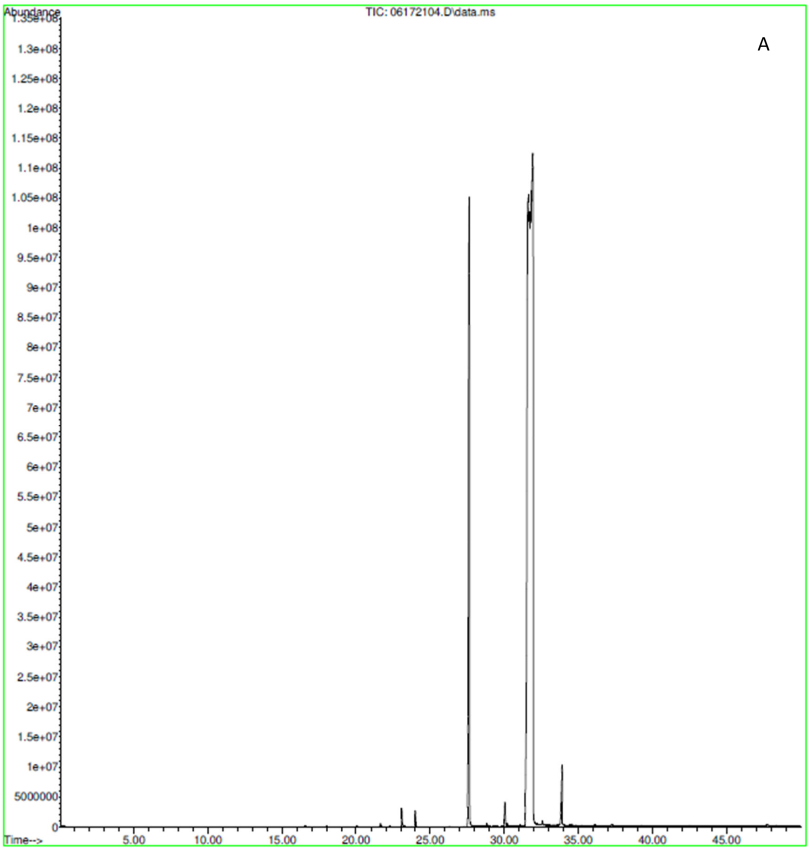

Unknown Spectrum based on Apex

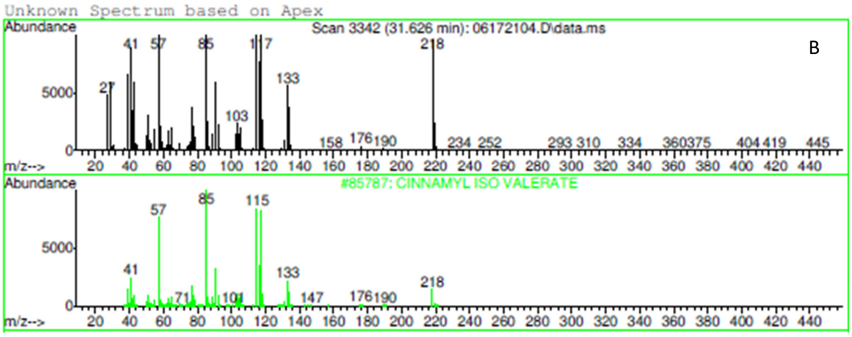

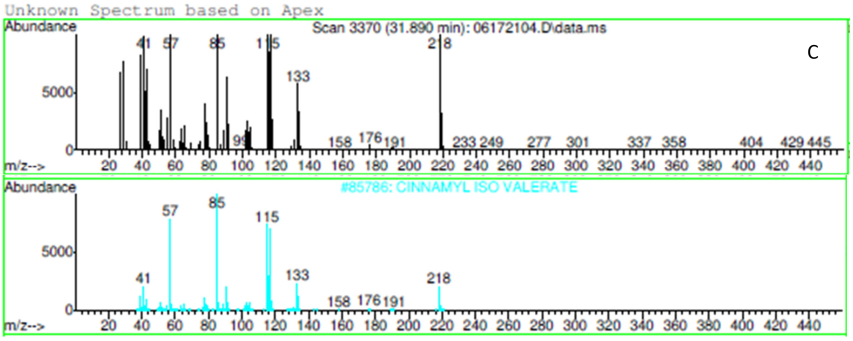

Figure S2. Chromatogram (A) and two sample mass spectra (B, C) for Methyl benzoate (CAS# 93-58-3) demonstrating poor chromatography. Injected after cinnamyl iso-valerate (CAS 140-27-2). Major peaks in this run (peaks 15 and 16 shown above) are identified as cinnamyl iso-valerate eluting late.

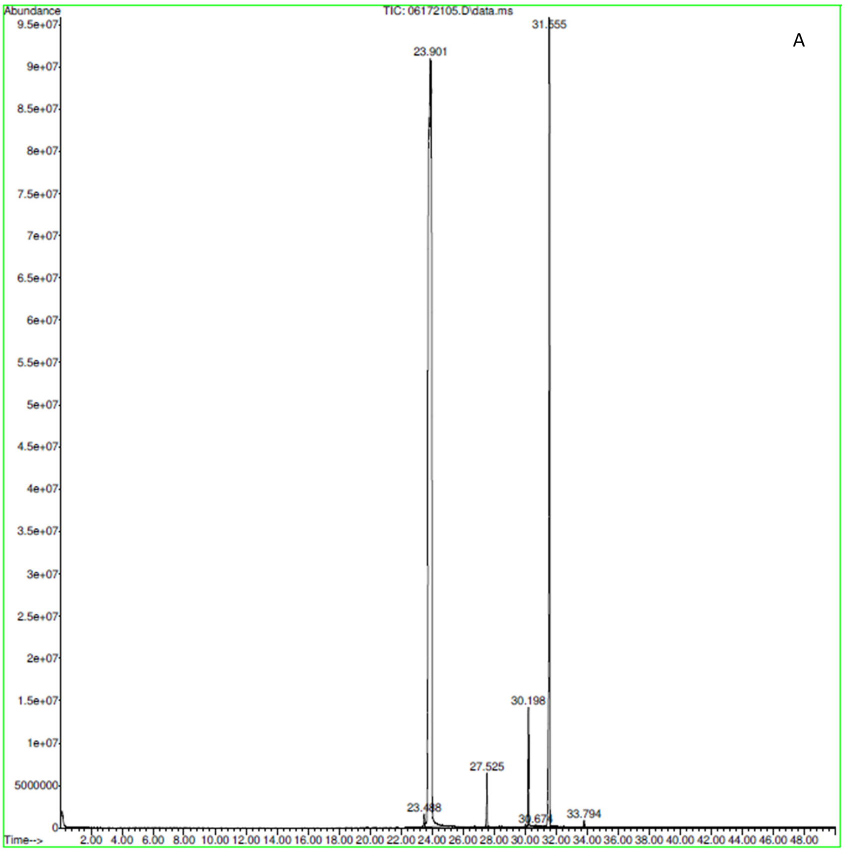

Sample Mass Spectra for Major Peak 2

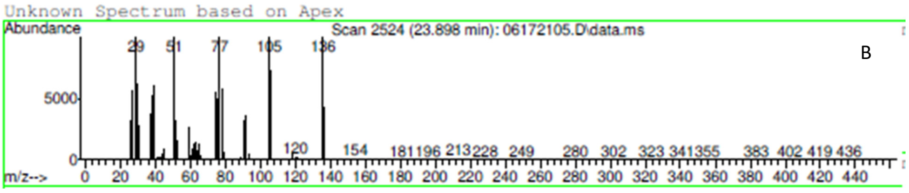

NIST Standard Mass Spectra

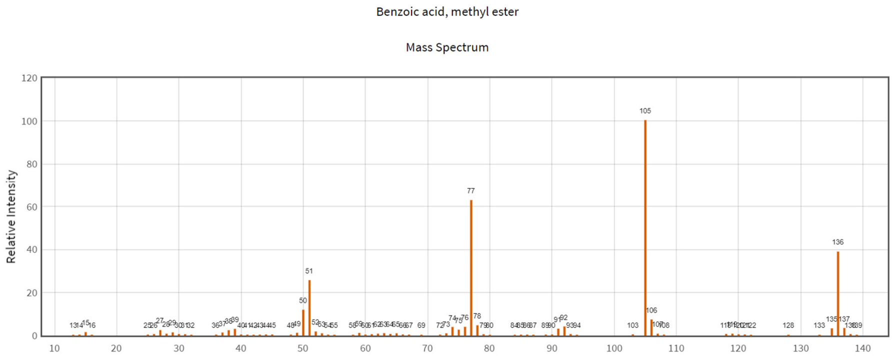

Peak 6 (RT 31.554) identified as remaining cinnamyl iso-valerate eluting

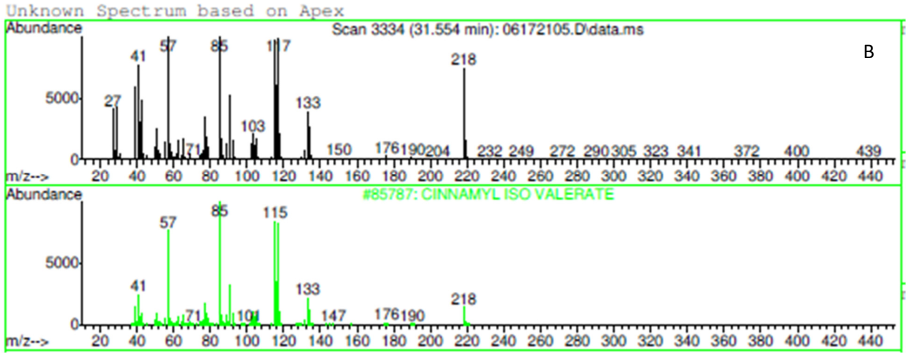

Figure S3. Chromatogram (A) and mass spectra (B) for Vinyl guaiacol (CAS# 7786-61-0) demonstrating poor chromatography. Injected after cinnamyl iso-valerate and methyl benzoate showing major peak 2 in this run is identified as methyl benzoate eluting late compared the NIST standard mass spectra. Second set of mass spectra shows Peak 6 (RT 31.554) was identified as remaining cinnamyl iso-valerate eluting with a library match score of 95.

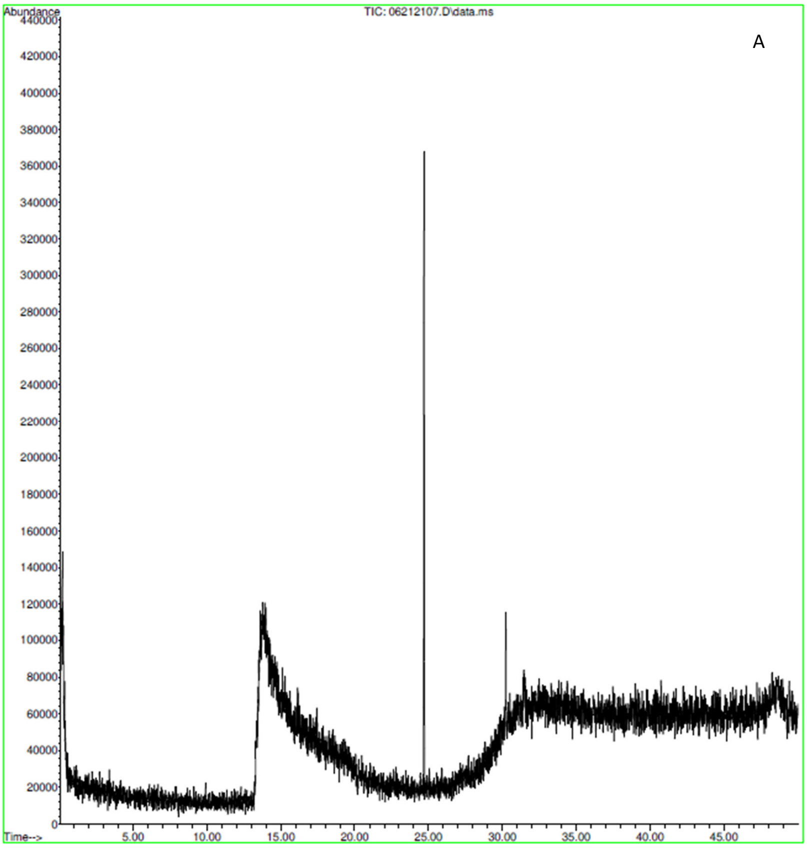

Figure S4. Chromatogram (A) for delta-3-carene (CAS# 13466-78-9) demonstrating poor chromatography with no real peaks.

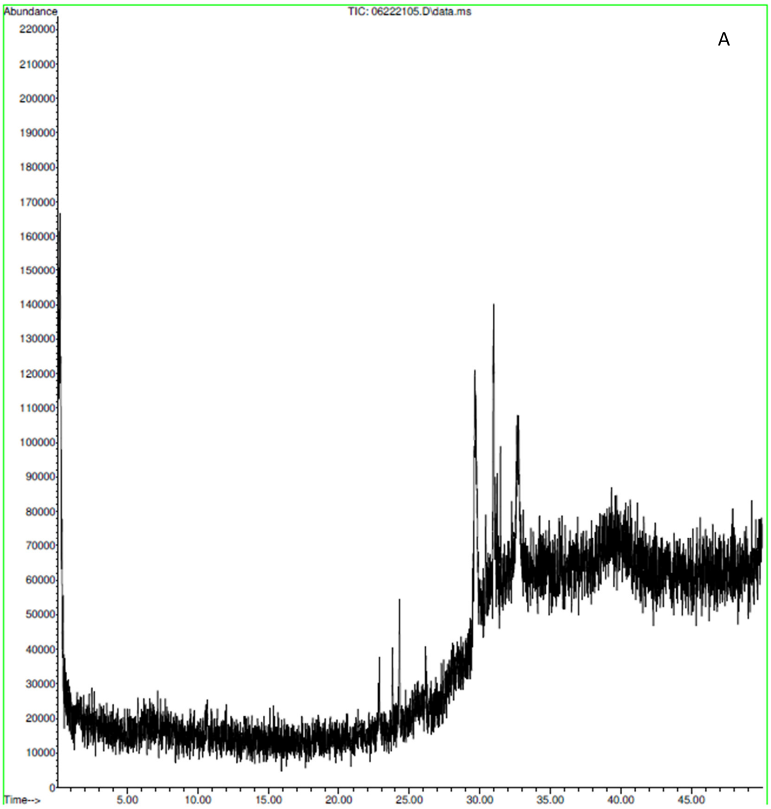

Figure S5. Chromatogram (A) for ethyl cinnamate (CAS# 103-36-6) demonstrating poor chromatography with no real peaks and eluting in the next run (Methyl-2 methyl-3-furyl disulfide injection).

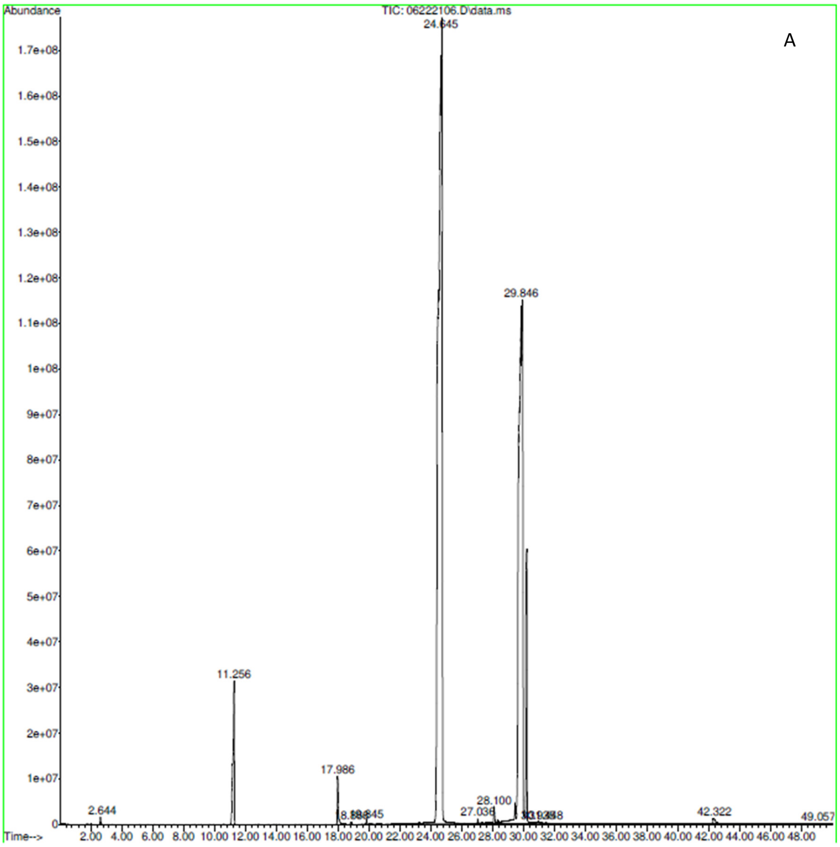

Sample Mass Spectra for Peak 9 (RT 29.849)

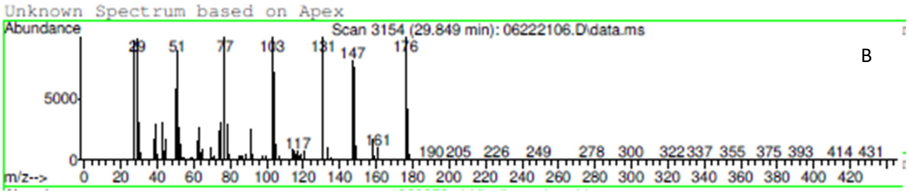

NIST Standard Mass Spectra

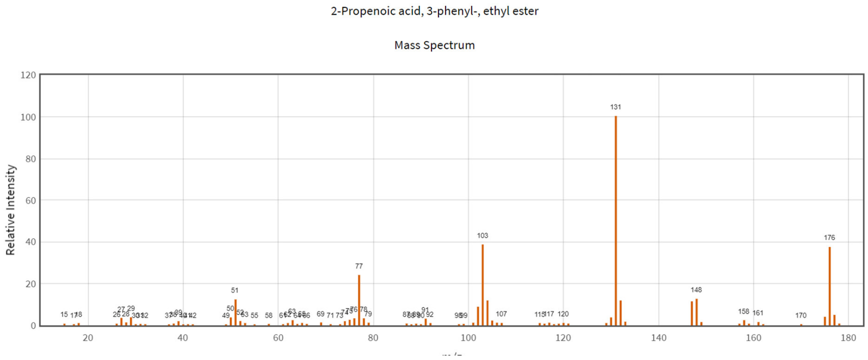

Figure S6. Chromatogram (A) and mass spectra (B) for Methyl-2 methyl-3-furyl disulfide (CAS# 65505-17-1) demonstrating poor chromatography. Injected after ethyl cinnamate (CAS# 103-36-6). Peak 9 (RT 29.849) misidentified due to saturation. Identified as ethyl cinnamate based on comparison to reference spectrum from NIST database.

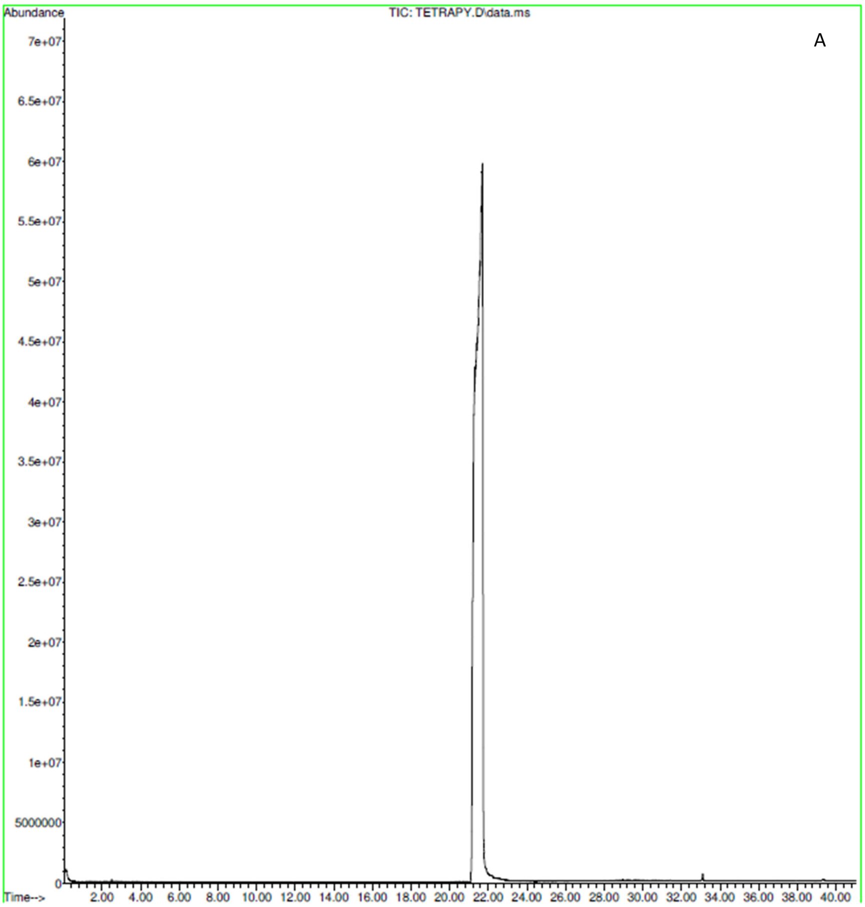

Abundance
TIC: TETRAPY.D\data.ms
A
7e+07
6.5e+07
6e+07
5.5e+07
5e+07
4.5e+07
4e+07
3.5e+07
3e+07
2.5e+07
2e+07
1.5e+07
1e+07
5000000
0
Time-->
2.00 4.00 6.00 8.00 10.00 12.00 14.00 16.00 18.00 20.00 22.00 24.00 26.00 28.00 30.00 32.00 34.00 36.00 38.00 40.00

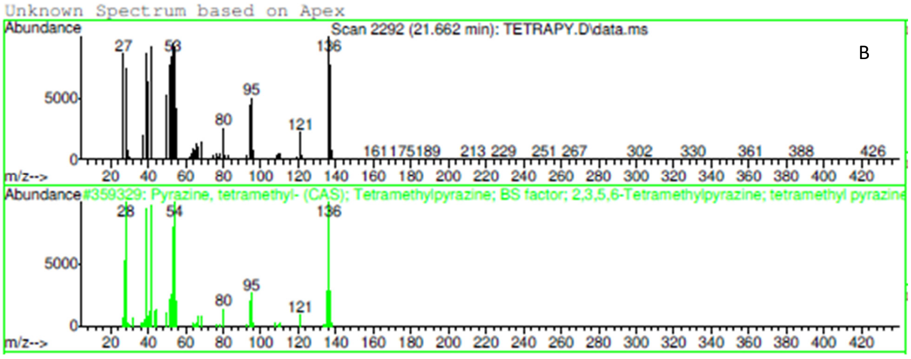

Figure S7. Chromatogram (A) and mass spectra (B) for Tetramethyl pyrazine (CAS# 1124-11-4) demonstrating good chromatography. Sample mass spectra (top) compared to library match mass spectra (bottom) are below the chromatogram.

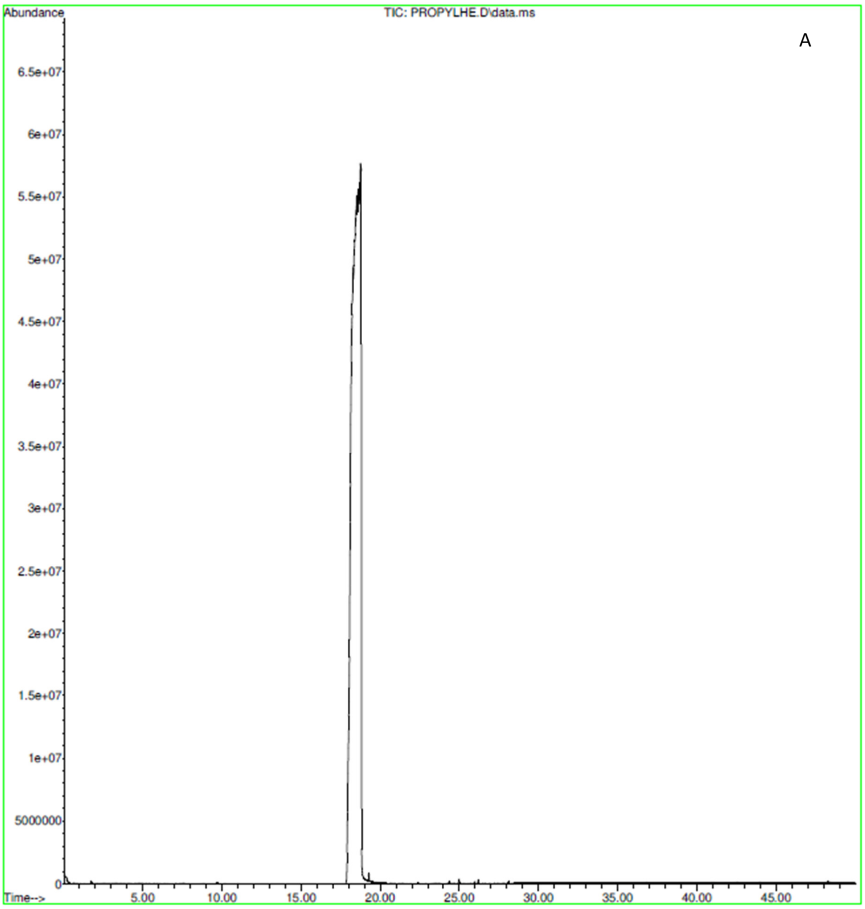
Abundance
TIC: PROPYLHE.D\data.ms
A
6.5e+07
6e+07
5.5e+07
5e+07
4.5e+07
4e+07
3.5e+07
3e+07
2.5e+07
2e+07
1.5e+07
1e+07
5000000
0
Time-->
5.00
10.00
15.00
20.00
25.00
30.00
35.00
40.00
45.00

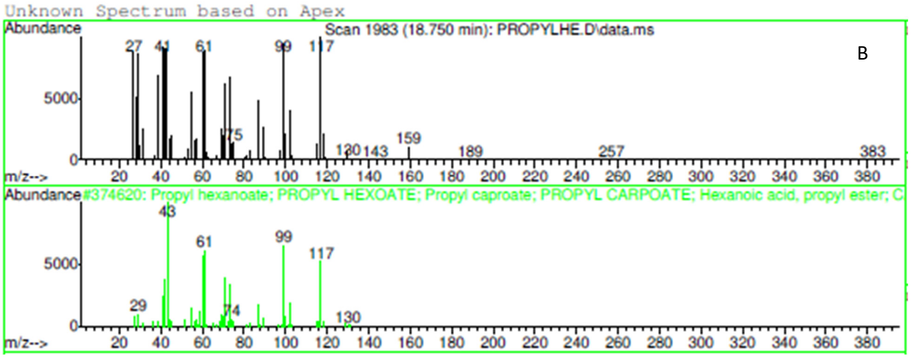

Figure S8. Chromatogram (A) and mass spectra (B) for Propyl caproate (CAS# 626-77-7) demonstrating good chromatography. Sample mass spectra (top) compared to library match mass spectra (bottom) are below the chromatogram.

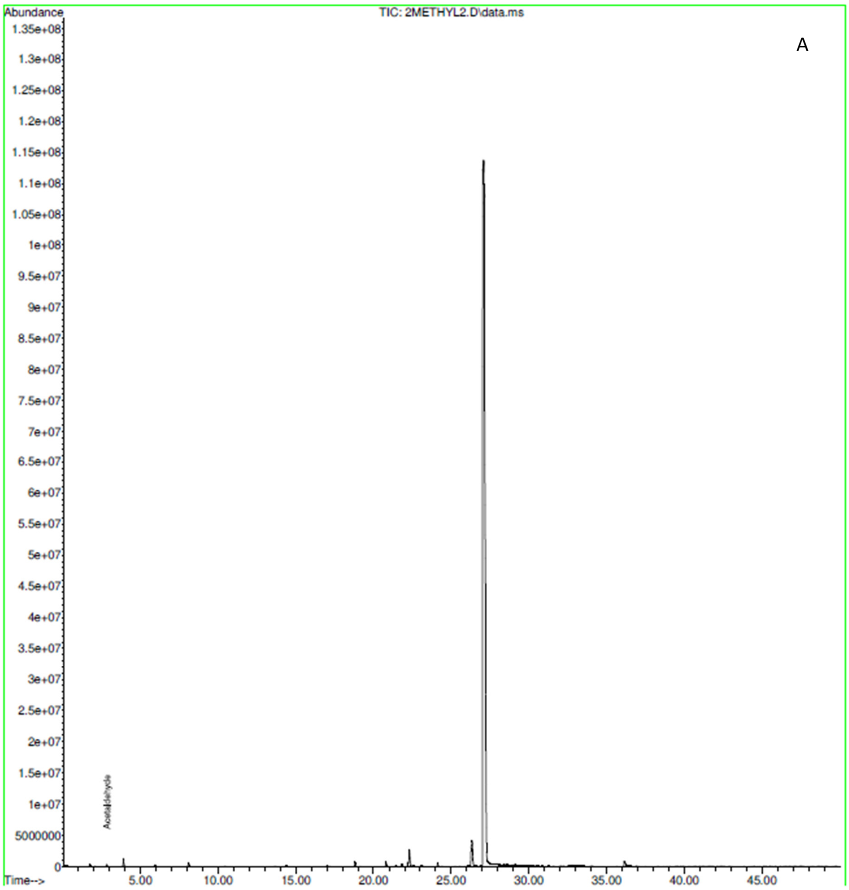

Sample Mass Spectra

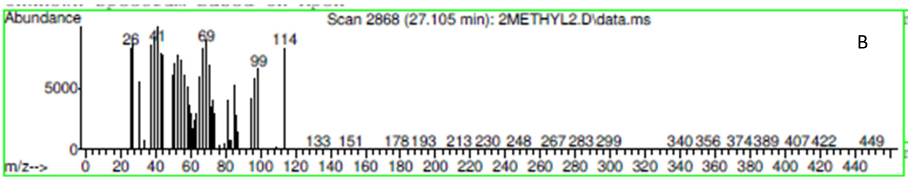

NIST Standard Mass Spectra

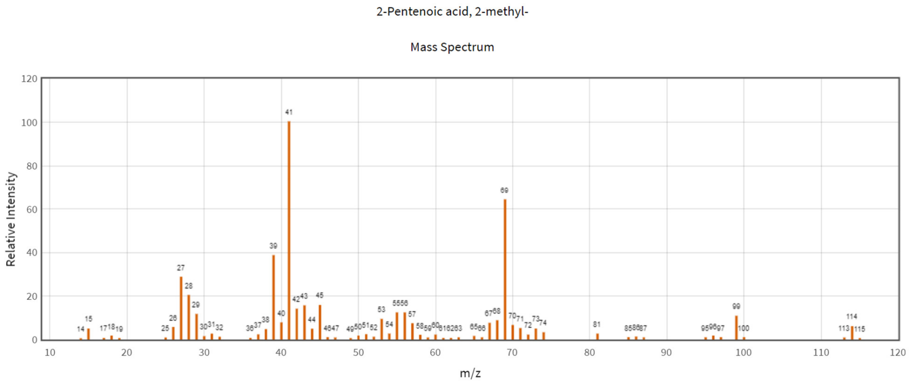

Figure S9. Chromatogram (A) and mass spectra (B) for 2-methyl-2-pentenoic acid (CAS# 3142-72-1) demonstrating good chromatography. Sample mass spectra (top) compared to NIST match mass spectra (bottom) are below the chromatogram. Peak 14 (RT 27.105 min) misidentified due to saturation. Identified as intact 2-methyl-2-pentenoic acid based on comparison to reference spectrum from NIST database.
